# Counteractive Effects of IL-33 and IL-37 on Inflammation in Osteoarthritis †

**DOI:** 10.3390/ijerph19095690

**Published:** 2022-05-07

**Authors:** Vikrant Rai, Matthew F. Dilisio, Farial Samadi, Devendra K. Agrawal

**Affiliations:** 1 Department of Translational Research, Western University of Health Sciences, Pomona, CA 91766, USA; vrai@westernu.edu; 2 Department of Orthopedic Surgery, Creighton University School of Medicine, Omaha, NE 68178, USA; matthew.dilisio@alegent.org; 3 Department of Biology, College of Arts and Sciences, University of Nebraska at Omaha, Omaha, NE 68182, USA; fsamadi@unomaha.edu

**Keywords:** osteoarthritis, inflammation, damage-associated molecular patterns, Interleukin-33, Interleukin-37, Toll-like receptors, matrix metalloproteinases, Macrophages

## Abstract

Osteoarthritis (OA) is a chronic inflammatory disease where pro-inflammatory cytokines, damage-associated molecular patterns (DAMPs), and macrophages play a crucial role. However, the interactive role of these mediators, the exact cause precipitating OA and definitive treatment for OA are not known yet. Moreover, the interactive role of interleukin (IL)-33 and IL-37 with other factors in the pathogenesis of OA has not been discussed elaborately. In this study, we analyzed the expression of IL-33 and IL-37 in human OA knee and hip joint cartilage tissues. The effect of increased DAMPs, IL-33, and IL-37 on IL-6, tumor necrosis factor (TNF)-α, toll-like receptors (TLRs), and matrix metalloproteinases (MMPs) expression was delineated using human normal and osteoarthritic chondrocytes. The effect of anti-inflammatory cytokine IL-37 on various mediators of inflammation in the presence of IL-33, rHMGB-1, and LPS was investigated to delineate the effects of IL-37. Further, the effects of blocking IL-33 downstream signaling and the effects of IL-33 and IL-37 on macrophage polarization were assessed along with examining the macrophage phenotypes in human OA cartilage tissues. The results of this study revealed increased expression of IL-33 in OA cartilage and that IL-33 increases IL-6, TNF-α, TLRs, and MMPs expression and favors phenotypic conversion towards the M1 phenotype, while IL-37 and blocking IL-33 receptor ST2 have opposite effects. Overall, the results suggest that blocking IL-33 and increasing IL-37 act synergistically to attenuate inflammation and might serve as potential therapeutics in OA.

## 1. Introduction

Osteoarthritis (OA), the most common form of arthritis involving mainly the knee and hip joint, is a wear-and-tear disease of the articular cartilage. OA is characterized by a limited range of motion, joint pain, tenderness, stiffness, crepitus, effusion, and joint inflammation [1,2]. Cartilage degeneration, osteophyte formation, subchondral bone sclerosis, weakness of the quadriceps muscles, and inflammation of the tendon, ligaments, and menisci are associated with OA [2,3]. Inflammation of the entire articular joint with an imbalance between the catabolic and anabolic cytokines and growth factors results in cartilage degradation and degeneration [3,4,5]. Inflammation of the joint may be caused by inflammatory immune cells, such as macrophages and the pro-inflammatory cytokines secreted from immune cells. Pro-inflammatory cytokines, including interleukin (IL)-6, IL-8, IL-15, IL-17, IL-18, IL-21, IL-1β, IL-33, and tumor necrosis factor (TNF)-α, and anti-inflammatory cytokines, such as IL-4, IL-10, IL-13, and IL-37, play an important role in the pathogenesis of arthritis [6,7,8,9,10,11,12]. These studies were conducted using synovial membrane in OA in animal models and the plasma levels in rheumatoid arthritis. Recently, few studies have reported the role of IL-33 and IL-37 in OA [13,14,15]; however, the interactive effect of IL-33 and IL-37 with other mediators of inflammation is lacking. In this study, we aimed to investigate IL-33 and IL-37 expression in human osteoarthritic cartilage Further, the effect of IL-33 and IL-37 and of blocking IL-33 downstream signaling on various mediators of inflammation involved in the pathogenesis of OA was investigated. An increased expression of damage-associated molecular patterns (DAMPs), including high-mobility group box-1 (HMGB-1), advanced glycation end products (AGEs), receptor for advanced glycation end products (RAGE), and cytokines, including TNF-α, IL-6, IL-1β, and IL-33 secreted through toll-like receptors (TLRs), is associated with cartilage damage [16,17,18,19,20,21]. Thus, we investigated the effect of recombinant (r)HMGB-1 on the mediators of inflammation, including IL-33, IL-6, TNF-α, matrix metalloproteinases (MMPs), TLRs, and anti-inflammatory cytokine IL-37 in human chondrocytes. Since IL-33 induces the lipopolysaccharide (LPS)-induced pro-inflammatory cytokines secretion whose expression by itself is regulated by LPS [22,23], we also evaluated the effects of LPS on the expression of IL-33, IL-6, TNF-α, MMPs, TLRs, and IL-37 in human chondrocytes.

Increased DAMPs cause increased secretion of pro-inflammatory cytokines, which further recruits immune cells such as macrophages, the most common immune cells involved in the pathogenesis of OA [3,24,25]. The presence of activated macrophages mediating progression of structural degeneration (CD163+ and CD14+) and pain (CD14+), and the use of soluble macrophage biomarkers as an indicator of inflammatory phenotypes suggest the critical role of macrophages in OA [26,27]. This is further supported by the presence of CD14+ cells and sCD14 in the degenerating cartilage and OA [3,28]. sCD14 instigates inflammation by activating monocyte/macrophage lineage cells, which further increase inflammation in degenerating cartilage [3,24]. The involvement of synovial macrophages in osteophyte formation and OA-related pathology [24] and the presence of macrophage inflammatory protein 3α (MIP-3α) and macrophage-derived pro-inflammatory cytokines in rheumatoid arthritis (RA) and OA further support the role of macrophages. [25]. Monocyte/macrophage transformation and M1: M2 polarization is regulated by LPS; cytokines, including interferon (IFN)-γ, IL-4, IL-13, IL-1β, and IL-10; and transforming growth factor (TGF)-β [29,30]. IL-33 polarizes monocytes towards classically (M1) and alternatively (M2) activated macrophages, while IL-37 promotes alternatively (M2) activated macrophages [31,32]. This suggests that the interaction between IL-33, IL-37, and macrophages might plays a role in inflammation-mediated cartilage degeneration, and this possible interaction has been investigated in this study [3]. Understanding the role of macrophage polarization is important because the role of M1 and M2 macrophage is context-dependent. M1 macrophages acts as pro-inflammatory mediators, while M2 macrophages resolve inflammation in inflammatory diseases involving innate and adaptive immune response, while the M2 macrophage has pro-tumoral and M1 has anti-tumor properties in tumor microenvironment [33,34].

The results of this study will support the notion that targeting inflammation by blocking IL-33 and increasing IL-37 in osteoarthritis will attenuate cartilage loss.

## 2. Materials and Methods

### 2.1. Patient Selection

**Figure 1:**
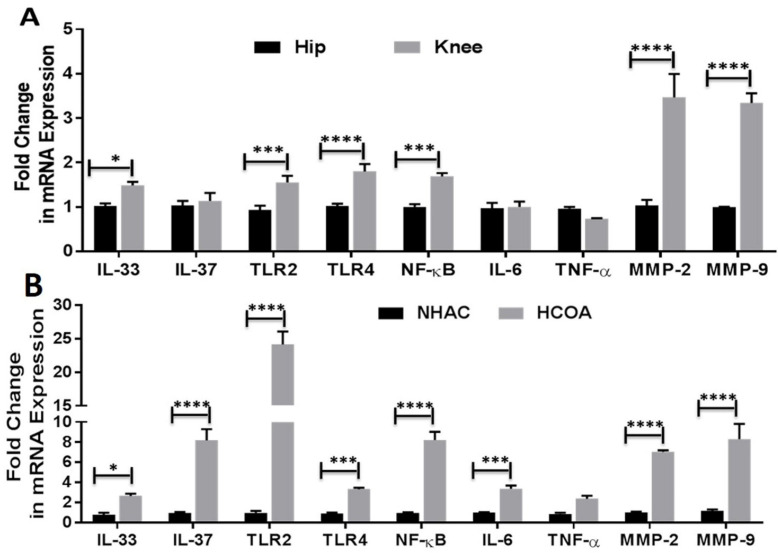
qRT-PCR for the mRNA expression of IL33, IL37, TLR-2, TLR-4, IL-6, TNFα, NF-κB, MMP2, and MMP9 in the osteoarthritic knee and hip joint cartilage, NHAC, and HCOA cells. qRT-PCR for the mRNA expression of IL-33, IL-37, TLR2, TLR4, IL-6, TNF-α, NF-κB, MMP2, and MMP9 in cartilage tissue of OA knee and hip joint (Panel (**A**)); qRT-PCR for the mRNA expression of IL-33, IL-37, TLR2, TLR4, IL-6, TNF-α, NF-κB, MMP2, and MMP9 in normal and OA chondrocytes (Panel (**B**)). HCOA, human chondrocytes osteoarthritic; IL, interleukin; MMP, matrix metalloproteinases; NHAC, normal human articular chondrocytes; NF-κB, nuclear factor kappa beta; OA, osteoarthritis; RT-PCR, real-time polymerase chain reaction; TLR, toll-like receptor; TNFα, tumor necrosis factor-alpha. Data are presented as mean ± SD from three separate experiments (N = 3). * *p* < 0.05, *** *p* < 0.001, and **** *p* < 0.0001.

Cartilage tissues were collected from discarded tissues after surgery anonymously from patients undergoing total knee or hip replacement due to severe osteoarthritis at Creighton University Medical Center (CUMC) and Immanuel Medical Center, Omaha, NE. The cartilage tissues from the discarded tissues were collected in a consistent manner from the edge of degenerating cartilages (as shown in Figure 1 panels A, B, and C of [18]). The research protocol for this prospective study was approved under the exempted category by the Institutional Review Board (IRB) of Creighton University. A total of 24 knee joint and 5 hip joint tissues were collected. Tissues were provided with demographic variables, including body mass index (BMI), age, gender, and race, in an anonymous manner without identifiers by a nurse not taking part in this study. The average age of the patients was 62.25 years (26 years to 78 years), and the average body mass index was 36.9 (15 to 69.48) for the knee-joint- and 70.8 years (60 years to 94 years) and 33.88 (23.68 to 41.5) for the hip-joint-replacement surgeries.

### 2.2. Tissue Acquisition and Processing

The discarded knee and hip joint tissues were collected and transported to the lab in UW (University of Wisconsin) solution and maintained at 4 °C. Articular cartilage tissues from the medial and lateral tibial condyles and femoral head near the area of cartilage loss and fat tissue attached to the surgical specimens were collected [18] and fixed in 4% formalin at room temperature. Formalin-fixed tissues were transversely sectioned at 2 mm after 24 h, followed by processing in Sakura Tissue Tek VIP Tissue Processor and paraffin-embedded. A microtome (Leica, Wetzlar, Germany) was used to obtain 5 μm thin sections, which were placed on the glass slides. Cartilage tissues were also used to prepare total RNA and cDNA for RT-PCR.

### 2.3. Cell Culture

Normal Human Articular Chondrocytes (NHAC) cells (HC1824, Lonza, Walkersville, MD, USA), and Osteoarthritic Human Chondrocytes (HCOA, 402OA-05a, Cell Applications Inc., San Diego, CA, USA) were cultured using complete chondrocyte basal media (CC-3217, 10% FBS + 1% penicillin-streptomycin) and chondrocyte growth medium (411–500, Cell Application), respectively, in a T25 flask in humidified 5% CO_2_ incubator at 37 °C. Second to third passages of NHAC and HCOA were used for all experiments. All cells were cultured in T75 or T25 flasks till 80–90% confluence and cell with 70–80% confluence were used for all experiments.

### 2.4. Hematoxylin and Eosin Staining of Specimen

Articular cartilage and fat tissues were stained with hematoxylin and eosin (HE) following the manufacturer’s standard protocol (Newcomer/supply). Stained tissues were scanned at 20× using an Olympus inverted microscope (Olympus BX51) with a scale bar of 200 μm. All the slides and scanned images were anonymously reviewed by two independent observers.

### 2.5. Immunostaining

The paraffin-fixed sections (cartilage and fat) were deparaffinized, rehydrated, and antigen retrieved before immunostaining following standard protocol in our laboratory. Briefly, after antigen retrieval, the slides were washed with PBS, blocked with 5% blocking buffer, and incubated with primary antibodies, including IL-33, IL-37, TLR-2, TLR-4, myeloid differentiation primary response 88 (MyD88), HMGB-1, RAGE, IL-6, TNFα, nuclear factor-kappa beta (NF-κB), phospho-NF-κB, MMP-2, MMP-9, collagen II, sox-9, chitinase-3 like protein 1, receptor for IL-33 (ST-2), cluster of differentiation (CD)14, CD86, CD206, CD162, and IL-10 (Supplementary Table S1), at titrated dilutions overnight at 4 °C. Alexa Fluor 594 (red) and Alexa Fluor 488 (green) (Invitrogen, Grand Island, NY, USA) at 1:500 dilutions were used as conjugated secondary antibodies. DAPI (4, 6-diamidino-2-phenylindole) was used to stain the nuclei. Negative controls were stained using IgG as a primary antibody without secondary antibodies and using only secondary antibodies while omitting the primary antibody. An Olympus inverted fluorescent microscope (Olympus BX51) was used to scan all stained sections. Fluorescence intensities for IL-33, IL-37, TLR-2, TLR-4, CD14, CD86, and CD206 in three different stained images from all tissues were measured using Image-J software, and mean fluorescence intensity (MFI) was calculated. Immunohistochemistry was performed using the peroxidase anti-peroxidase method using a secondary antibody conjugated to horseradish peroxidase (HRP) using the Vectastain ABC kit following standard protocol in laboratory.

### 2.6. Immunofluorescence (IF) of Chondrocytes

Approximately 3000 cultured chondrocytes were plated in each chamber of the chamber slide and cultured overnight. The following day, the cells were fixed with 5% formalin for 10 min followed by treatment with 0.1% triton for 15 min and washed three times 5 min each with PBS. The chondrocytes were incubated with primary antibodies (Supplementary Table S1) at 4 °C overnight and washed three times with PBS followed by incubation with secondary antibodies for 30 min at room temperature. After washing slides three times 5 min each, DAPI was used to stain the nucleus. The stained cells were scanned at 20× with an Olympus inverted fluorescent microscope (Olympus BX51). Fluorescence intensities for IL-33, IL-37, TLR-2, and TLR-4 in 15 random chondrocytes from each image were measured using Image-J software, and mean fluorescence intensity (MFI) was calculated.

### 2.7. RNA Isolation, cDNA Synthesis, and Real-Time PCR

Trizol reagent (Sigma, St Louis, MO, USA) was used to isolate total RNA from cartilage tissues, and cultured chondrocytes following manufacturer’s instructions. The yield of total RNA was quantified using Nanodrop (Thermo Scientific, Rockford, IL, USA), and cDNA was synthesized using Improm II reverse transcription kit (Promega, Madison, WI, USA). The quantitative real-time PCR (qRT-PCR) was performed in triplicate using SYBR Green Master Mix (BioRad #1708882) using a Real-time PCR system (CFX96, BioRad Laboratories, Hercules, CA, USA). The primers for different genes (Supplementary Table S2) were purchased from Integrated DNA Technologies (Coralville, IA, USA). RT-PCR was run with an initial denaturation at 95 °C for 5 min, 40 cycles of 30 s at 95 °C, 30 s at 55–60 °C based on primer annealing temperatures, and 30 s at 72 °C followed by melting curve analysis. The folds change in mRNA expression relative to controls was determined using 2^−^^^^ct^ method after normalization to housekeeping gene GAPDH.

### 2.8. Stimulation and Inhibition Studies

To study the effect of IL-33, IL-37, rHMGB-1, and LPS on various inflammatory mediators, NHAC cells were cultured in a T-75 flask, and approximately 200,000 cells were plated in each well of the 6-well plate. After 80–90% confluence, cells were treated with recombinant human IL-33 cytokine (25 ng/mL), recombinant human IL-37 cytokine (25 ng/mL), recombinant human HMGB-1 (500 ng/mL), and LPS (100 ng/mL) for 24 h. The concentrations of IL-33 and IL-37 were decided after titration studies with different doses. After the treatment fold change in mRNA expression, IL-33, IL-37, TLR-2, TLR-4, IL-6, TNF-α, NF-κB, MMP-2, and MMP-9 was analyzed. In another experiment, NHAC cells were stimulated with recombinant human IL-33 cytokine (25 ng/mL), recombinant human HMGB-1 (500 ng/mL), and LPS (100 ng/mL) for 2 h followed by treatment with recombinant human IL-37 cytokine (25 ng/mL) for 24 h. Total RNA was extracted, and cDNA was prepared and subjected to qRT-PCR for the mRNA expression of IL-33, IL-37, TLR-2, TLR-4, IL-6, TNF-α, NF-κB, MMP-2, and MMP-9. The folds change in mRNA expression relative to controls was determined using 2^−^^^^ct^ method after normalization to GAPDH (Supplementary Table S2).

### 2.9. Blocking Studies to Investigate the Effect of Blocking ST2 Receptor

To study the effect of blocking IL-33 receptor ST2 on the mediators of inflammation, approximately 200,000 NHAC cells plated in a 6-well plate were cultured to 80–90% confluence. The cells were treated with an anti-ST2 specific blocking antibody for 1 h followed by recombinant human IL-33 cytokine (25 ng/mL) along with matched control. qRT-PCR for change in mRNA expression of IL-33, TLR-2, TLR-4, IL-6, TNF-α, NF-κB, RAGE, HMGB-1, MMP-2, and MMP-9 was carried out, and the results were analyzed compared to control after standardizing with GAPDH.

### 2.10. Cell Culture and Macrophage Polarization Studies

Human monocytes (THP-1 cells) were cultured and propagated in a T25 flask using RPMI complete media (5% fetal bovine serum + 1% penicillin-streptomycin) in a humidified incubator with 5% CO_2_ at 37 °C. At 80% confluence, 200,000 cells were plated in each well of a 6-well plate and treated with Phorbol 12-myristate 13-acetate (PMA) for 48 h to convert monocytes (THP-1) to macrophage. After the cells became attached to the flask surface and transformed into macrophages, cells were treated with IL-33 (10 ng.mL) and IL-37 (10 ng/mL) for 6 days, changing the media every second day. The concentrations of IL-33 and IL-37 were decided after titration studies with different doses. After 6 days, the cells were trypsinized and subjected to flow cytometry for analyzing the positively stained cells for CD86 and CCR7 for M1 macrophage and CD206, CD163, and IL-10 for M2a, M2b, and M2c macrophages, respectively, using the standard protocol. Briefly, the cells were washed with PBS4 (PBS+ 4% fetal bovine serum) and centrifuged (300× *g* 10 min) followed by incubation with primary antibodies for CD86, CCR7, CD206, CD163, and IL-10 (Supplementary Table S1) for 45 min at a concentration of 10^6^ cells/10μL/. Cells were centrifuged (300× *g* 10 min), and 500 mL of FACS-fix (PBS:10% formaldehyde in 3:2) was added to the cells after removing supernatant. Isotype for each fluorochrome was used for negative control. OneComp eBeads (eBioscience 01-1111-42, Thermo Fische, Waltham, MA, USA) with fluorescently conjugated antibody was used for positive control. Live cells were gated using FSC/SSC. For threshold, a gate was first set from the forward-versus side-scatter dot-plot of all events in the area corresponding to the size and granularity of macrophages with spleen as a reference. LIVE/DEAD Fixable Violet Dead Cell Stain Kit (L34964, Thermo Fischer Scientific, Waltham, MA, USA) was used for sorting live cells. Flow cytometry was performed on a BD FACSAria Fusion (https://www.bdbiosciences.com/en-us/products/instruments/flow-cytometers/research-cell-sorters/bd-facsaria-fusion (accessed on 12 January 2022)) and cell populations were analyzed using Flow-Jo (v10) software (TreeStar). The average count of positively stained cells from three separate experiments was analyzed for significance.

### 2.11. Statistical Analysis

Data are presented as mean ± SD (N = 3 in each group). Data were analyzed using one-way ANOVA with Bonferroni correction and Student’s *t*-test for significance between the two groups. A value of *p* < 0.05 (* *p* < 0.05, ** *p* < 0.01, *** *p* < 0.001, and **** *p* < 0.0001) was considered statistically significant.

## 3. Results

### 3.1. Expression of IL-33, IL-37, TLRs, IL-6, TNF-α, and MMPs in Human OA Cartilage and Chondrocytes

**Figure 2:**
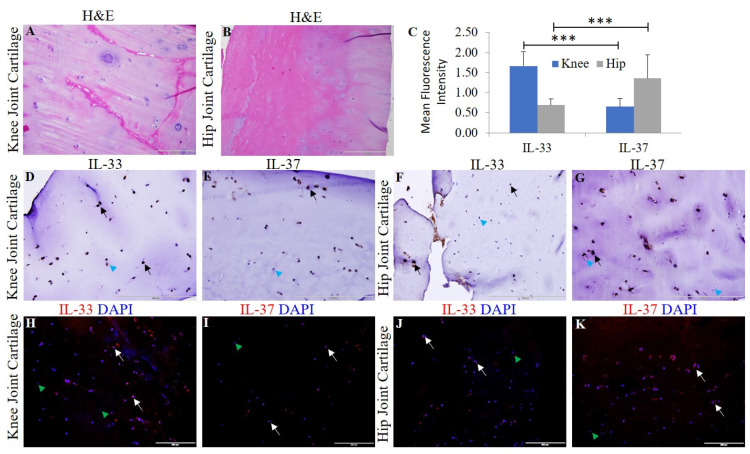
H&E and immunostaining for IL-33 and IL-37 in the osteoarthritic knee and hip joint cartilage. H&E for IL-33 in knee joint cartilage (panel (**A**)) and hip joint cartilage (panel (**B**)); immunohistochemistry for IL-33 and IL-37 in the knee (panels (**D**,**E**)) and in hip joint cartilage (panels (**F**,**G**)), respectively. Immunofluorescence for IL-33 (panels (**H**,**J**)) and IL-37 (panels (**I**,**K**)). MFI for IL-33 and IL-37 (panel (**C**)) in human OA cartilage. DAPI, 4, 6-diamidino-2-phenylindole; IL, interleukin; MFI, mean fluorescence intensity; OA, osteoarthritis. These are the representative images from all patients included in this study. Data are presented as mean ± SD (N = 3). A *p*-value < 0.05 was considered as significant; *** *p* < 0.001. Black arrows indicate positive and blue arrowheads indicate negative staining in panels (**D**–**G**) for IL-33 and IL-37. White arrows indicate positive and green arrowheads indicate negative staining in panels (**H**–**K**) for IL-33 and IL-37.

**Figure 3:**
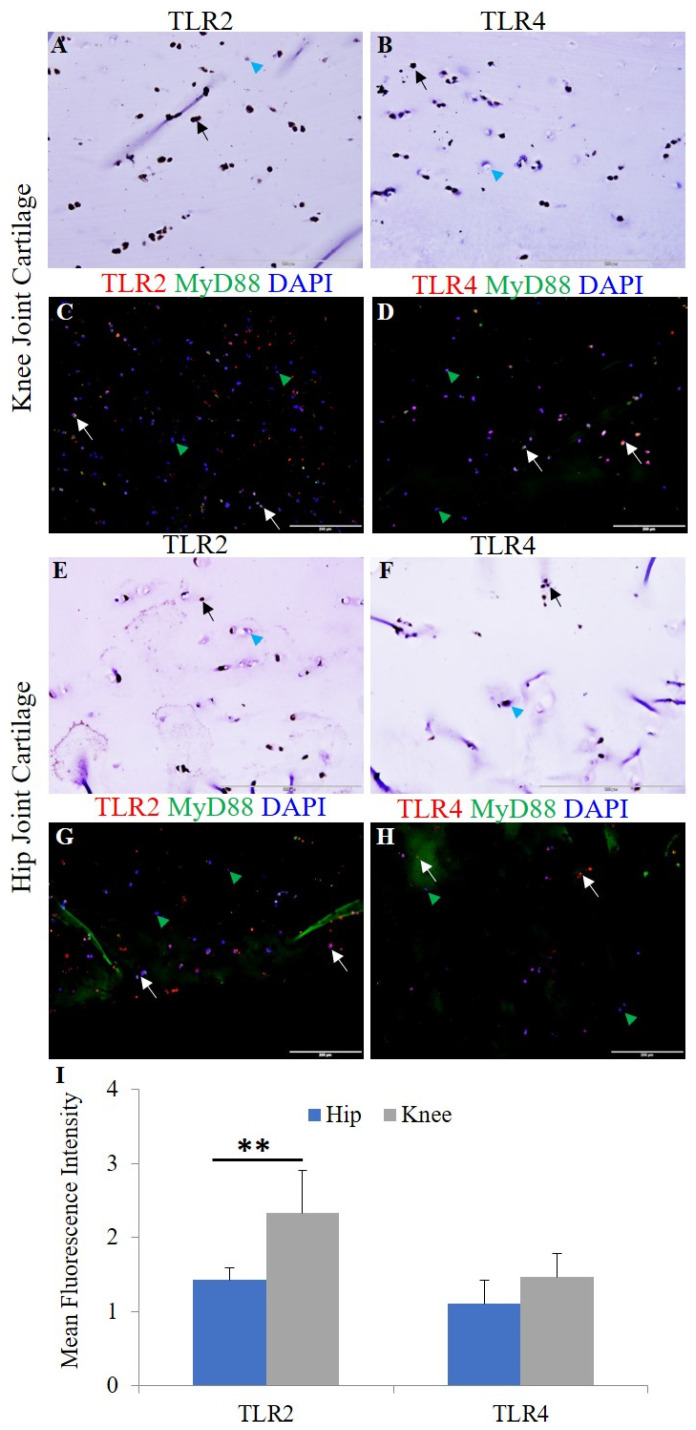
Immunofluorescence for TLR2/MyD88 and TLR4/MyD88 in the osteoarthritic knee and hip joint cartilage. Immunohistochemistry for TLR2 (panels (**A**,**E**)) and TLR4 (panels (**B**,**F**)), immunofluorescence for TLR2 (panels (**C**,**G**)), and TLR4 (panels (**D**,**H**)). MFI for TLR2 and TLR4 in human cartilage tissue (panel (**I**)). DAPI, 4,6-diamidino-2-phenylindole; MFI, mean fluorescence intensity; MyD88, myeloid differentiation primary response 88; TLR, toll-like receptor. These are the representative images from all patients included in this study. Data are presented as mean ± SD (N = 3). A *p*-value < 0.05 was considered as significant; ** *p* < 0.01. Black and white arrows indicate positive and blue and green arrowheads indicate negative staining in panels (**A**–**H**) for IL-33 and IL-37.

Chondrocyte-specific markers collagen II, Sox-9, and chitinase-3 like protein 1 (Supplementary Figure S1) and H&E staining were used to characterize the OA knee and hip joint cartilage tissues [18]. The normal (NHAC) and osteoarthritic (HCOA) chondrocytes were characterized by chondrocyte-specific markers (Supplementary Figure S2). qRT-PCR and immunofluorescence revealed positive expression for IL-33, IL-37, TLR-2, TLR-4, NF-κB, IL-6, TNF-α, MMP-2, and MMP-9 at the gene (Figure 1) and protein level (Figure 2 and Figure 3 and Supplementary Figures S3–S5). qRT-PCR analysis revealed significantly increased mRNA expression of IL-33, TLR-2, TLR-4, NF-κB, MMP-2, and MMP-9 in OA knee joint compared to the hip joint cartilage (Figure 1A) and significantly increased mRNA expression of IL-33, IL-37, TLR-2, TLR-4, IL-6, NF-κB, MMP-2, and MMP-9 in HCOA compared to NHAC cells (Figure 1B). IF revealed a higher expression of IL-33 in the knee joint compared to the hip joint cartilage and higher expression of IL-37 in the hip joint compared to the knee joint cartilage (Figure 2). The immunopositivity for TLR-2 and TLR-4 (Figure 3), IL-6 and TNF-α (Supplementary Figure S3), MMP-2 and MMP-9 (Supplementary Figure S4), and NF-κB, and pNF-κB (Supplementary Figure S5) was higher in the knee joint compared to the hip joint cartilage. IL-37 (Supplementary Figure S6), TLR-2 and TLR-4 (Supplementary Figure S7), IL-6 and TNF-α (Supplementary Figure S8), MMP-2 and MMP-9 (Supplementary Figure S9), and NF-κB, and pNF-κB (Supplementary Figure S10) immunopositivity was higher in HCOA compared to NHAC. The immunopositivity for IL-33 was higher in NHAC compared to HCOA (Supplementary Figure S6). Nuclear staining for IL-33 while both nuclear and cytoplasmic staining for IL-37, TLR-2, TLR-4, IL-6, TNF-α, NF-κB, pNF-κB, MMP-2, and MMP-9 was noted on immunofluorescence.

### 3.2. IL-33, Recombinant (r)HMGB-1, and LPS Upregulate the mRNA Expression of IL-33, TLRs, IL-6, TNF-α, NF-κB, MMPs, HMGB-1, and RAGE

**Figure 4:**
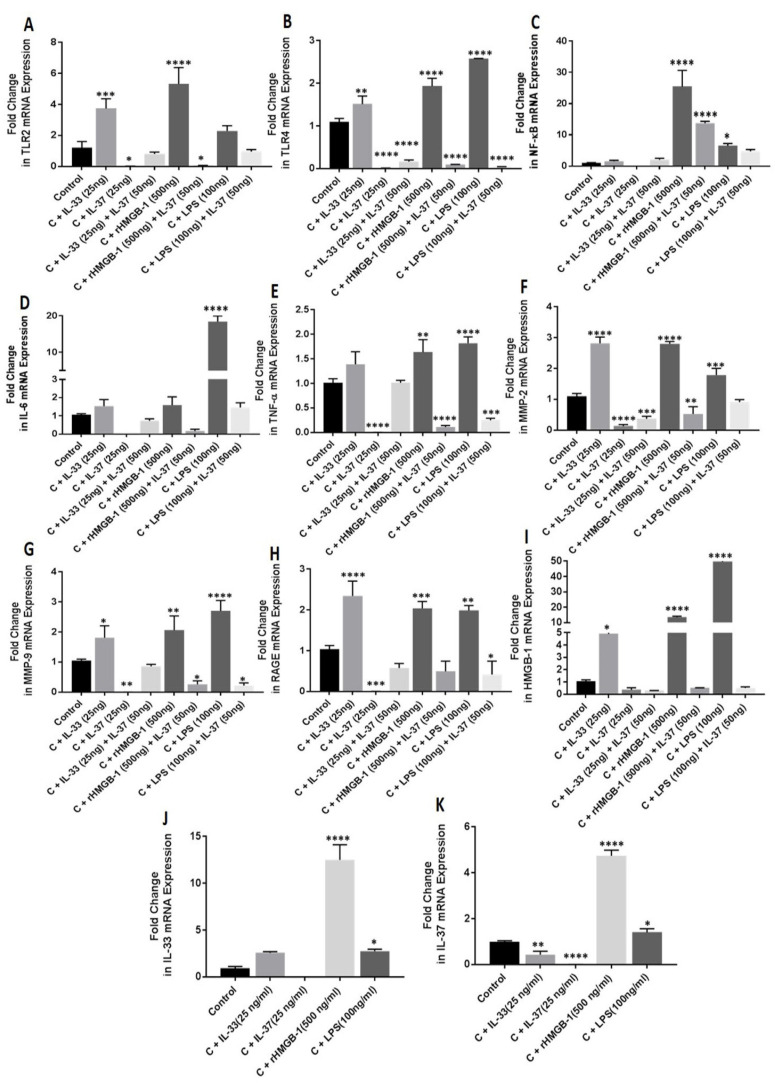
RT-PCR for the fold change in mRNA expression of TLR-2, TLR-4, IL-6, TNFα, NF-κB, MMP2, MMP9, RAGE, HMGB1, IL33, and IL37 in normal and IL-33-, IL-37-, rHMGB1-, and LPS-treated NHAC cells. TLR2 (Panel (**A**)), TLR4 (Panel (**B**)), NF-κB (Panel (**C**)), IL-6 (Panel (**D**)), TNFα (Panel (**E**)), MMP-2 (Panel (**F**)), MMP-9 (Panel (**G**)), RAGE (Panel (**H**)), HMGB1 (Panel (**I**)), IL-33 (Panel (**J**)), and IL-37 (Panel (**K**)). C, control; HCOA, human osteoarthritic chondrocytes; HMGB1, high-mobility group box 1; IL, interleukin; LPS, lipopolysaccharides; MMP, matrix metalloproteinases; NHAC, normal human articular chondrocytes; NF-κB, nuclear factor kappa beta; rHMGB1, recombinant HMGB1; RTPCR, real-time polymerase chain reaction; RAGE, receptor for advanced glycation end-products; TLR, toll-like receptor; TNFα, tumor necrosis factor-alpha. Data are presented as mean ± SD (N = 3). * *p* < 0.05, ** *p* < 0.01, *** *p* < 0.001, and **** *p* < 0.0001.

qRT-PCR analysis of IL-33- (25 ng/mL), rHMGB-1- (500 ng/mL), and LPS- (100 ng/mL) treated NHAC cells showed increased mRNA expression of IL-33, TLR-2, TLR-4, IL-6, TNF-α, NF-κB, MMP-2, MMP-9, HMGB-1, and RAGE in the cells treated for 24 h compared to the untreated cells (Figure 4).

### 3.3. IL-37 Attenuates mRNA Expression of IL-37, TLRs, IL-6, TNF-α, NF-κB, MMPs, HMGB-1, and RAGE

qRT-PCR analysis of IL-37- (25 ng/mL) treated NHAC cells showed decreased mRNA expression of IL-37, TLR-2, TLR-4, IL-6, TNF-α, NF-κB, MMP-2, MMP-9, HMGB-1, and RAGE in the cells treated for 24 h compared to the untreated cells (Figure 4).

### 3.4. IL-33 Downregulates While rHMGB-1 and RAGE Upregulate the mRNA Expression of IL-37

qRT-PCR analysis of IL-33- (25 ng/mL), rHMGB-1- (500 ng/mL), and LPS- (100 ng/mL) treated NHAC cells showed increased mRNA expression of IL-37 with rHMGB-1 and LPS while decreased mRNA expression of IL-37 with IL-33 (Figure 4) in the cells treated for 24 h compared to the untreated cells.

### 3.5. IL-37 Attenuates the Stimulatory Effect of IL-33, rHMGB-1, and LPS on the mRNA Expression of TLR-2, TLR-4, IL-6, TNF-α, NF-κB, MMP-2, MMP-9, HMGB-1, and RAGE

To evaluate the effect of IL-37 in presence of IL-33, cDNA synthesized from NHAC cells treated with IL-37 (50 ng/mL) followed by IL-33 (25 ng/mL), rHMGB-1 (500 ng/mL), and LPS (100 ng/mL) for 24 h was subjected to qRT-PCR. The results showed attenuation of the mRNA expression of TLR-2, TLR-4, NF-κB, IL-6, TNF-α, MMP-2, MMP-9, HMGB-1, and RAGE in NHAC cells treated with IL-37 followed by IL-33, rHMGB-1, and LPS compared to NHAC cells treated with IL-33 (25 ng/mL), rHMGB-1 (500 ng/mL), and LPS (100 ng/mL) individually and the untreated NHAC cells (Figure 4).

### 3.6. Anti-ST2 Antibody Blocks the Effect of IL-33

**Figure 5:**
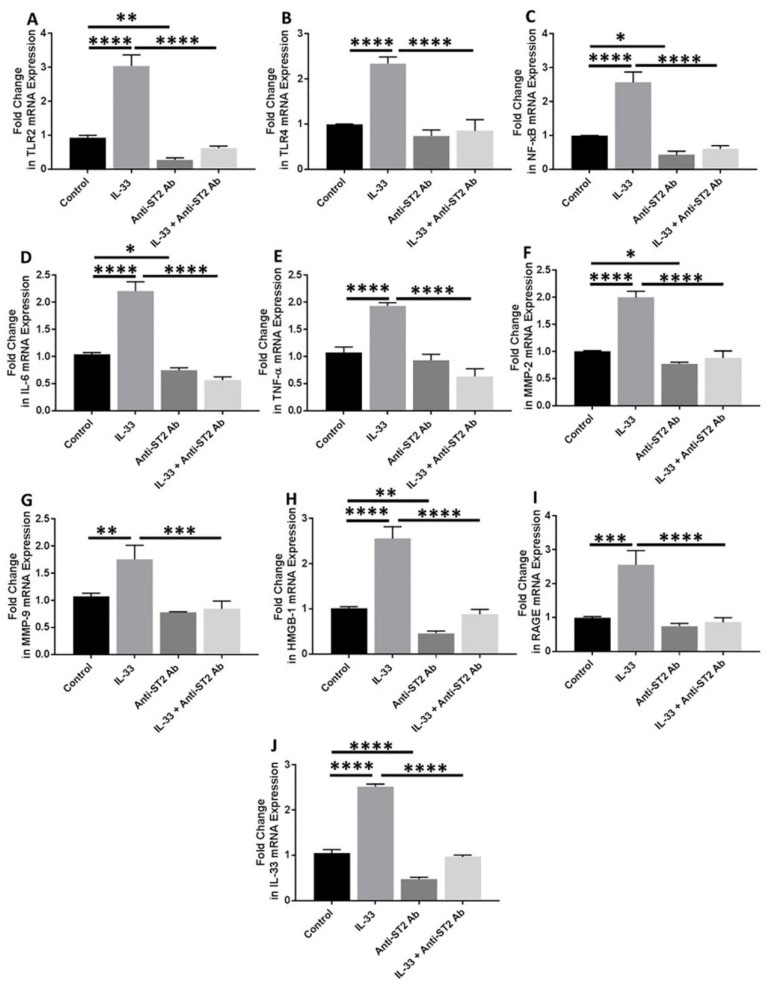
RT-PCR for the fold change in mRNA expression of IL-33, TLR-2, TLR-4, IL-6, and TNFα, NF-κB, MMP2, MMP9, RAGE, and HMGB1 in anti-ST2 antibody-treated NHAC cells. Folds change in mRNA expression of TLR2 (panel (**A**)), TLR4 (panel (**B**)), NF-κB (panel (**C**)), IL-6 (panel (**D**)), TNFα (panel (**E**)), MMP-2 (panel (**F**))MMP-9 (panel (**G**)), HMGB-1 (panel (**H**)), RAGE (panel (**I**)), and IL-33 (panel (**J**)) in presence of IL-33, anti-ST2 antibody, and IL-33 in presence of anti-ST2 antibody. C, control; HMGB1, high-mobility group box 1; IL, interleukin; MMP, matrix metalloproteinases; NHAC, normal human articular chondrocytes; NF-κB, nuclear factor kappa beta; RTPCR, real-time polymerase chain reaction; RAGE, receptor for advanced glycation end-products; TLR, toll-like receptor; TNFα, tumor necrosis factor-alpha. Data are presented as mean ± SD (N = 3). * *p* < 0.05, ** *p* < 0.01, *** *p* < 0.001, and **** *p* < 0.0001.

Anti-ST2 antibody was used to block the IL-33 receptor (ST2) to check the effect of blocking downstream signaling of IL-33. qRT-PCR analyses of the cDNA prepared from the cells treated with anti-ST2 antibody followed by IL-33 showed significantly attenuated mRNA expression of IL-33, TLR-2, IL-6, TNF-α, and MMP-9 and decreased mRNA expression of TLR-4, NF-κB, RAGE, and HMGB-1 (Figure 5). These results suggest that ST2 receptor blocking results in inhibition of the effect of IL-33 on its downstream signaling.

### 3.7. Significantly Higher Expression of M2a Macrophage in OA Knee and Hip Joint Cartilage

**Figure 6:**
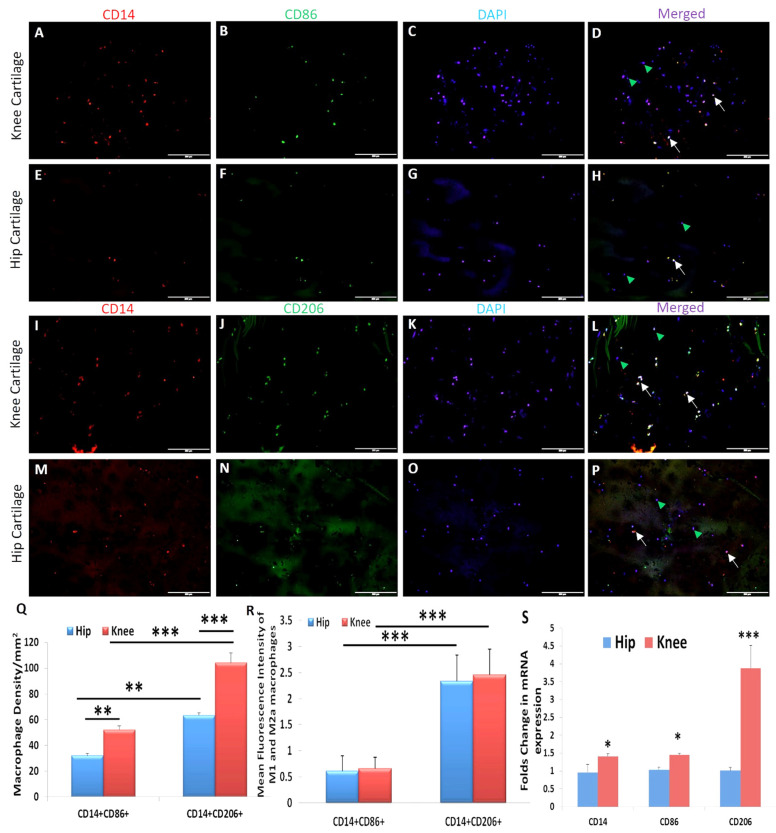
Immunofluorescence and RT-PCR for gene and protein expression of CD14, CD86, and CD206 in the osteoarthritic knee and hip joint cartilage. CD14 (Panels (**A**,**E**,**I**,**M**)), CD86 (Panels (**B**,**J**)), CD206 (Panels (**F**,**N**)), DAPI (Panels (**C**,**G**,**K**,**O**)), merged (Panels (**D**,**H**,**L**,**P**)), macrophage density in the knee and hip joint OA cartilage (Panel (**Q**)); mean fluorescence intensity of CD14, CD86, and CD206 in the knee and hip joint OA cartilage (Panel (**R**)); and mRNA expression of CD14, CD86, and CD206 in the knee and hip joint OA cartilage (Panel (**S**)). These are the representative images of all patients included in this study. Data are presented as mean ± SD (N = 3). * *p* < 0.05, ** *p* < 0.01, *** *p* < 0.001 CD, cluster differentiation; DAPI, 4′,6-diamidino-2-phenylindole; RT-PCR, real-time-polymerase chain reaction. All images were scanned at 200 μm.

Immunopositivity for CD14 (macrophage), CD86 (M1 macrophage), and CD206 (M2a macrophage) (Figure 6) while minimal immunopositivity (Supplementary Figure S11) for CD163 (M2b macrophage) and IL-10 (M2c macrophage) was noted in post-surgical OA knee and hip joint cartilages using dual immunofluorescence. The immunopositivity for CD14+ CD86+ cells (M1 macrophages) and CD14+ CD206+ cells (M2a macrophages) in the knee joint cartilage was significantly higher compared to the hip joint cartilage. Knee joint cartilage tissues showed significantly higher macrophage density and mean fluorescence intensity for M1 and M2a macrophages compared to the hip joint cartilage. The mRNA expression for CD14, CD86, and CD206 was significantly higher in the knee joint cartilage compared to the hip joint cartilage (Figure 6).

### 3.8. IL-33 Favors M1 While IL-37 Favors M2 Macrophage Phenotype

**Figure 7:**
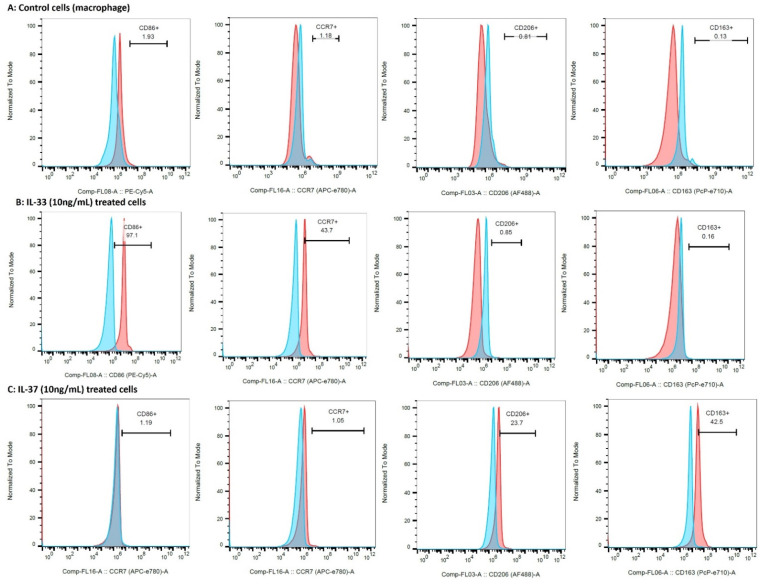
Flow cytometry for macrophage polarization and dose-dependent effect of IL-37. Control macrophages (panel (**A**)), IL-33-treated macrophages (panel (**B**)), and IL-37-treated macrophages (panel (**C**)).

**Figure 8:**
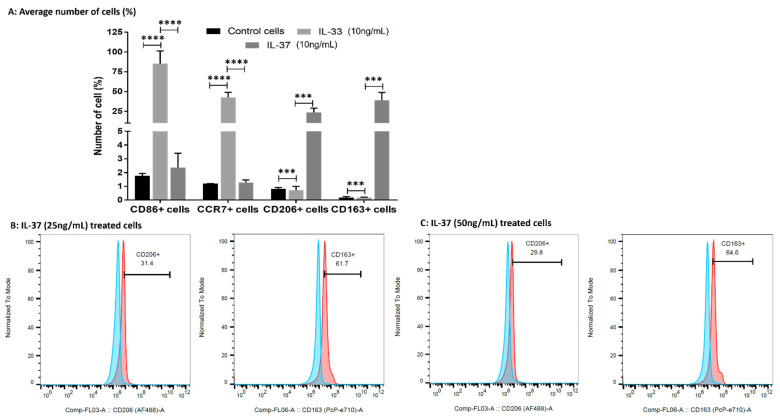
Average percentages of positive cells after treatment with IL-33 and IL-37 compared to control cells (panel (**A**)) and dose-dependent effect of IL-37 on macrophage polarization (panels (**B**,**C**)). Blue color- isotype control and red-antibody stained. Data are presented as mean ± SD (N = 3), *** *p* < 0.001, **** *p* < 0.0001.

The flow-cytometry results of the IL-33- and IL-37-treated macrophages showed the predominance of CD86+ (M1; 85.1 ± 16.26%) and CCR7+ (M1; 42.47 ± 6.44%) cells in IL-33- (10 ng/mL) treated cells while the predominance of CD206+ (M2a; 23.43 ± 5.41%) and CD163+ (M2b; 39.07 ± 9.81%) cells in IL-37 (10 ng/mL) treated cells compared to untreated control cells (Figure 7, Supplementary Table S3). These results suggest the effect of IL-33 and IL-37 on macrophage polarization. Flow cytometry results also revealed a dose-dependent effect on the polarization of M2a and M2b macrophages with an increased number of M2a- and M2b-positive cells with the higher doses of IL-37 (25 and 50 ng/mL). There was higher percentage of CD206+ (M2a; 33.8% and 31.3%) and CD163+ (M2b; 64.9% and 60.8%) cells with the higher concentrations of IL-37 (Figure 8, Supplementary Table S3).

## 4. Discussion

The results of this study showed positive gene and protein expression for IL-33, IL-37, TLR-2, TLR-4, IL-6, and TNF-α, NF-κB, HMGB-1, RAGE (hereafter proinflammatory mediators), MMP-2 and MMP-9 (hereafter pro-damage mediators), and M1 and M2 macrophages in osteoarthritic knee and hip joint cartilage. The positive expression of pro-inflammatory and pro-damage mediators in the knee and hip joint cartilage supports the hypothesis of crucial role of these mediators in the pathogenesis of OA [3,20,21,35]. This is further supported by the similar expression profile of these mediators in HCOA compared to NHAC. These finding suggest our hypothesis of the crucial role of IL-33 and IL-37 in OA. The mRNA expression of IL-37 was higher in the knee joint cartilage; however, the protein expression of IL-37 was higher in the hip joint cartilage (Figure 1 and Figure 2). IL-37 is an anti-inflammatory cytokine, and differential gene and protein expression in cartilage tissues may be due to differential expression of DAMPs [18] and IL-33 in the knee and hip joint cartilage. Further, a higher expression of these mediators in the knee joint suggests higher levels of inflammation and the extent of cartilage damage in the knee joint. This might be the reason behind the lower expression of IL-37 and higher incidences of OA in the knee joint compared to the hip joint [18,19,36]. The extent of articular cartilage damage may influence the expression levels of these mediators of inflammation, and to investigate this, we analyzed the thickness of the articular cartilage from the lateral and medial condyle of the tibia and the head of the femur on H&E staining [18]. The average ± SD values of 1.33 ± 0.38 and 1.36 ± 0.48 for the thickness of the knee and hip joint cartilage respectively indicate no significant difference in the extent of damage of the cartilage tissue and suggest that inflammation is due to increased inflammatory mediators in the knee joint compared to hip joint, and thickness of the damaged cartilage has not affected the results of this study. This conclusion is also supported by the fact that all the issues were collected in a consistent manner from a terminal disease requiring joint replacement.

A positive expression of IL-33 in the knee and hip joint cartilage in this study might be due to increased expression of DAMPs in the knee and hip joint [18,20,37]. An increased mRNA expression of IL-33, IL-37, TLR-2, TLR-4, NF-κB, IL-6, TNF-α, MMP-2, MMP-9, RAGE, and HMGB-1 (Figure 4) in NHAC treated with rHMGB-1 suggests the potential role of HMGB-1 in increasing the expression of these mediators and supports the findings of this study. Since HMGB-1 has its effects through TLRs [38], the presence (Supplementary Figure S7) and colocalization of TLRs with HMGB-1 (Supplementary Figure S12) indicate the presence of HMGB-1 and TLRs in chondrocytes and cartilage. These results were further supported by the stimulation studies showing increased mRNA expression of TLRs in NHAC with rHMGB-1. RAGE is a ligand for HMGB-1 and increased mRNA expression of RAGE with rHMGB-1 in NHAC cells and the co-localization of HMGB-1 with RAGE support the notion of increased expression of inflammatory mediators with HMGB-1 via TLRs and RAGE [16,17]. The accumulation of DAMPs (HMGB-1), increased secretion of IL-33, and TLRs in the knee and hip joint cartilage suggests a vicious cycle causing cartilage loss [16,17]. Further, increased expression of NK-κB, IL-6, and TNF-α leading to an increased expression of MMP-2 and MMP-9 with rHMGB-1 suggest HMGB-1 as a key player in upregulating inflammatory mediators during cartilage loss [20,21].

IL-33 is a ligand for IL-1 family receptor T1/ST2 (ST2L) and mediates its biological effects through activation of nuclear factor kappa beta (NF-κB) [39]. IL-33 is secreted upon cell death, cell necrosis or apoptosis, or under stress and mechanical injury [37]. IL-33 activates TLR-2 and TLR-4 in a MyD88-dependent manner and exhibits a pro-inflammatory potential by increasing the production of pro-inflammatory chemokines and cytokines. TLR-4 activation also mediates secretion of IL-33, thus leading to a continuum of inflammation. LPS-mediated increased secretion of pro-inflammatory cytokines is augmented by IL-33, and this suggests a synergistic effect of the LPS-IL33-TLR axis [8,22,40,41]. IL-33 and IL-37 was expressed differentially in the knee and hip joint with a positive expression of IL-33 in both the knee and hip joint cartilage. IL-33 expression was more in the knee joint compared to the hip joint, and IL-37 expression was more in the hip joint compared to the knee joint. This might be due to differential expression of DAMPs (HMGB-1 and RAGE) and more direct load, damage, and shear stress on the knee joint compared to the hip joint. This might also be due to the nature of the tissue collected for this study (severe OA requiring knee/hip joint replacement) [18,36]. These findings suggest that an imbalance between the detrimental (IL-33) and beneficial (IL-37) factors in the cartilage may have potentially caused continued cartilage loss and associated with a higher prevalence of knee joint OA (3.8%) compared to hip joint OA (0.85%) [42].

Increased expression of IL-33, TLR-2, TLR-4, NF-κB, IL-6, TNF-α, RAGE, HMGB-1, MMP-2, and MMP-9 with IL-33 and decreased expression of with IL-37 in NHAC suggests the counteractive effect of IL-33 and IL-37. Further, increased expression of these mediators with IL-33, rHMGB-1, and LPS and downregulation with IL-37 suggests the pro-inflammatory effect of IL-33 and HMGB-1 and anti-inflammatory effect of IL-37 in the cartilage [6,20,21,35,43]. These in-vitro results corroborate immunostaining and RT-PCR findings in the OA cartilage tissue of the knee and hip joints. The effect of IL-37, as found in this study, is supported by the fact that the defense mechanism of the body, in response to increased inflammation, mediates the secretion of IL-37 to avert the detrimental effect of inflammation and attenuate the secretion of pro-inflammatory cytokines through inhibition of NF-κB [10,44,45,46]. Thus, attenuated expression of these mediators with IL-37 suggests the therapeutic potential of IL-37 in OA to downregulate inflammation and cartilage loss. Further, attenuation of the effects of IL-33, rHMGB-1, and LPS on the expression of IL-33, TLR-2, TLR-4, NF-κB, IL-6, TNF-α, RAGE, HMGB-1, MMP-2, and MMP-9 with IL-37 support the potential of the therapeutic role of IL-37 in OA. The positive expression of IL-33 in the knee and hip joint cartilage and increased expression of IL-33 in HCOA compared to NHAC suggest IL-33 as a potential therapeutic target to attenuate chronic inflammation and cartilage loss [13,47]. This notion is supported by the decreased expression of IL-33, TLR-2, TLR-4, NF-κB, IL-6, TNF-α, RAGE, HMGB-1, MMP-2, and MMP-9 in NHAC with anti-ST2 antibody (ST2 receptor of IL-33, Figure 5) [7,8,13,20,21,22,39,41]. TLR activation and increased pro-inflammatory cytokines with LPS mediate inflammation in the joint cartilage [21,48,49,50]. The increased mRNA expression of IL-33, TLRs, IL-6, TNF-α, and MMPs in LPS-treated cells and their attenuation with IL-37 in the presence of LPS suggests the attenuating effect of IL-37 and its anti-inflammatory and beneficial role in OA. These results suggest that decreasing IL-33 expression, blocking IL-33 downstream signaling, and increasing IL-37 may act synergistically to attenuate inflammation and cartilage loss. A recent development in using 3D scaffolds with mesenchymal stem cells for cartilage repair and the reports of IL-37 in protecting these stem cells and decreasing proteoglycan loss suggest incorporating anti-IL-33 and IL-37/pro-IL-37 strategies for better therapeutic outcome in OA [14,15,51].

The role of macrophage-mediated inflammation in the OA cartilage and synovial membrane [3,24,25] is supported by the differential expression of various chemokines and their receptors, including a C-C chemokine receptor (CCR) 2, C-C Motif Chemokine Ligand (CCL) 3, CCL5, C-X-C chemokine receptor (CXCR) 1, CXCR2, and C-X-C motif ligand (CXCL) 8 in the synovial membrane [52]. Immunopositivity of CD14, CD86, and CD206 in this study suggests the presence of macrophages and CD14+ chondrocytes in the OA cartilage [53,54]. Pro-inflammatory macrophages (M1) predominate during the early phase and anti-inflammatory macrophages (M2) predominate during the late phase of inflammation. Significantly higher mRNA expression, macrophage density, immunoreactivity, and mean fluorescence intensity (MFI) of CD14+CD206+ cells suggest the predominance of M2 macrophage compared to M1 macrophage in OA knee and hip joint cartilage (Figure 6) and corresponds to the terminal disease of the collected samples. The process of initiation, development, and cessation of inflammation is regulated by the transformation of macrophages into different phenotypes termed macrophage polarization [29,55]. Macrophage polarization towards an M1 and M2 phenotype is regulated by IL-33 and IL-37 in addition to LPS, interferon (IFN)-γ, IL-4, IL-13, IL-1β, IL-10, and transforming growth factor (TGF)-β. IL-33 favors both M1 and M2 phenotype, and IL-37 favor M2 phenotype in a context-dependent manner [30,32,33,34,56,57]. Since sequential delivery of cytokines enhances vascularization of the bone scaffold through macrophage polarization [30], we investigated the effects of IL-33 and IL-37 on macrophage polarization. The results showed M1 predominance with IL-33 and M2 predominance with IL-37 (Figure 7 and Figure 8). These results suggest that sequential delivery of anti-IL-33 factors and IL-37 cytokines might be beneficial in attenuating inflammation and cartilage loss. However, presence of the predominant M2 macrophage in the cartilage of OA knee and hip joint raise the question regarding why, with predominant M2 macrophages, there is continued cartilage degeneration. This might be due to a very high propensity of M1 macrophage inducing chronic inflammation for a long time, delayed M2 macrophage polarization, an imbalance between IL-33 and IL-37, and differential expression of IL-33 and IL-37 in the knee and hip joint due to shear stress resulting in prolonged inflammation causing a continued cartilage degeneration in the study population. This suggests that an early polarization of the infiltrating macrophage with a predominant M2 population via sequential release of cytokines and growth factors using a scaffold might help in better vascularization, decreased inflammation, and preventing cartilage loss [30]. This strategy looks feasible because macrophage polarization attenuates inflammation in various inflammatory diseases, including RA [29,30,55,58,59], and sequential delivery of cytokines and growth factors may also improve the survival of mesenchymal stem cells being used for cartilage repair [51,60]. Another concern was the presence of macrophages in the cartilage, which is an avascular structure. Thus, we evaluated the presence of chemokine factors responsible for the migration of macrophages in the cartilage tissue. A positive expression of chemokine receptors CCR2 and CCR7, chemokine ligands CCL3 and CCL5 (Supplementary Figure S13), angiogenic factors VEGF, and endothelial cell marker CD31 (Supplementary Figure S14) suggests the presence of factors contributing to the recruitment of macrophages. The minimal immunopositivity for VEGF and CD31 as observed in this study might be due to the end-stage disease of the cartilage due to the fissuring and flanking effect [61,62,63]. The recruitment of the macrophages or CD14+ cells in the cartilage from synovium might be due to differential binding and/or expression of these chemokines on synovium. The macrophage migration may also be facilitated by the damaged extracellular matrix of the cartilage. The presence of M1 and M2a macrophage in a ratio of 2:1 in both knee and hip joint fat tissue (collected from supra- and infra-patellar area and around the hip joint; Supplementary Figure S15) in this study suggests the presence of inflammation in the joint fat, which in turn plays a crucial role in the weakness of muscle, tendon, and ligament via inflammation and fatty infiltration [3].

## 5. Conclusions

**Figure 9:**
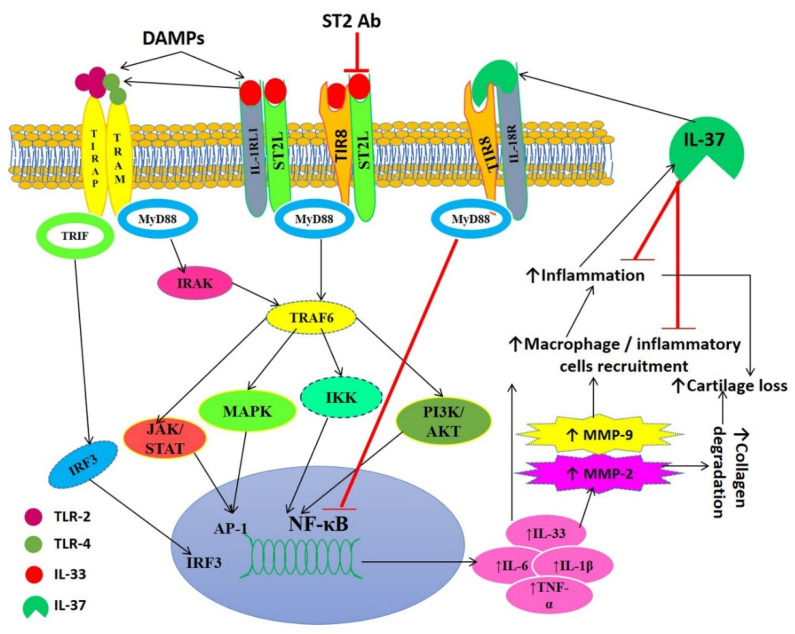
Schematic representation of IL-33-mediated inflammation, the role of IL-37, and translational approach of blocking IL-33 downstream signaling. Damage-associated molecular patterns (DAMPs) mediate the release of interleukin (IL)-33. IL-33 activates the toll-like receptors (TLR2 and TLR4) and transcription factor nuclear factor kappa beta (NF-κB). This leads to increased secretion of pro-inflammatory cytokines, including IL-6, IL-1β, IL-33, and tumor necrosis factor (TNF) α, resulting in the activation of matrix metalloproteinases and recruitment of inflammatory cells. This results in increased inflammation and degradation of the cartilage matrix leading to the progression of cartilage degeneration and osteoarthritis (OA). Inflammation also leads to the secretion of IL-37 that in turn suppresses inflammation by decreasing the recruitment of inflammatory cells and inhibiting NF-κB. The blocking of IL-33 receptor ST2 will result in the attenuation of TLR activation and decreased secretion of proinflammatory cytokines. Thus, blocking the IL-33 receptor, the use of IL-33 inhibitors and IL-37 may be potential therapeutic strategies. Ab, antibody; AKT, Protein kinase B (PKB); IRF3, interferon regulatory transcription factor; IKK, I-kappa-B kinase; IRAK, interleukin-1 receptor-associated kinase 1; IL-1R1, interleukin 1 Receptor Type 1; JAK/STAT, Janus kinase/signal transducer and activator of transcription; MAPK, mitogen-activated protein kinases; MyD88, myeloid differentiation primary response 88; PI3K, Phosphatidylinositol-4,5-bisphosphate3-kinase; TRIF, TIR domain-containing adapter-inducing interferon-β; TIR8, toll-interleukin 1 receptor 8; TRAF6, TNF receptor-associated factor 6.

Overall, the results of this study correlate an association between DAMPs and an increased expression of pro-inflammatory and pro-damage factors in OA cartilage from human patients. The counteractive effects of IL-33 and IL-37 on the mediators of inflammation and attenuation of the effects of IL-33, rHMGB-1, and LPS by IL-37 suggest the potential role of using IL-37 and anti-IL-33 strategies in combination to have a better therapeutic outcome. This is also supported by the fact that OA is a multifactorial disease, and thus, multiple factors should be targeted. Additionally, the decreased expression of inflammatory mediators with anti-ST2 antibody and IL-37 in this study support the use of IL-37 and anti-IL-33 strategies in OA to attenuate inflammation and cartilage damage synergistically [13,14,15,64]. Further, the predominance of M2 phenotype and attenuation of the effect of IL-33, rHMGB-1, and LPS on mediators of inflammation with IL-37 suggest the therapeutic potential of IL-37 (Figure 9). In this study, we compared different findings between the knee and hip joint to elucidate the differential expression and to correlate with the kinematics of the knee and hip joint with different degrees of OA. In addition, the different mechanics in different joints and well-known changes in gene expression in cartilage from different origins need a comparative analysis to elucidate the differential expression of factors responsible for OA to design personalized therapeutics.

## 6. Limitations of the Study

This study highlighted the importance of blocking IL-33/ST2 and enhancing IL-37 to attenuate inflammation and cartilage degeneration using in-vitro studies supported by results from human osteoarthritic cartilage. A limited number of human hip joints and the non-availability of normal cartilage are the limitations. Another limitation is the limited amount of osteoarthritic cartilage tissue from human patients. Further, the lack of information related to confounding factors, including the medical and personal history, occupational history of the study subjects may have confounded the results of this study.

## Data Availability

All data are included in the manuscript and as Supplementary Material.

## Supplementary Materials

The following are available online at https://www.mdpi.com/article/10.3390/ijerph19095690/s1, Figure S1: Immunofluorescence for the characterization of chondrocytes in OA cartilage; Figure S2: Immunofluorescence for the characterization of chondrocytes in NHAC and HCOA cells; Figure S3: Immunofluorescence for IL-6 and TNFα in OA knee and hip joint cartilage; Figure S4: Immunofluorescence for MMP2 and MMP9 in OA cartilage and fat tissue; Figure S5: Immunofluorescence for NF-κB and phospho NF-κB in OA cartilage; Figure S6: Immunofluorescence for IL-33 and IL-37 in NHAC and HCOA cells; Figure S7: Immunofluorescence for TLR2/MyD88 and TLR4/MyD88 in NHAC and HCOA cells; Figure S8: Immunofluorescence for IL-6 and TNFα in NHAC and HCOA cells; Figure S9: Immunofluorescence for MMP2 and MMP9 in NHAC and HCOA cells; Figure S10: Immunofluorescence for NF-κB NHAC and HCOA cells; Figure S11: Immunofluorescence for M2b (CD163) and M2c (IL-10) macrophages in the knee and hip joint cartilage; Figure S12: Immunofluorescence staining for the co-localization of TLR-2, TLR-4, and RAGE with HMGB-1; Figure S13: Immunofluorescence of CCR2, CCL3, CCL5, and CCR7 in osteoarthritic human cartilage; Figure S14: Immunopositivity for vasculoendothelial growth factor (VEGF) and CD31 in human osteoarthritic cartilage; Figure S15: Immunofluorescence of macrophage in OA osteoarthritic knee and hip joint fat; Table S1: Primary antibodies used for immunofluorescence and flow cytometry; Table S2: Forward and reverse primer sequence of the gene of interest used for RT-PCR analysis; Table S3: Flow-cytometry analysis of IL-33- and IL-37-treated macrophages (number (%) of positive cells).
