# Supplementary material for: Counteractive Effects of IL-33 and IL-37 on Inflammation in Osteoarthritis
Source: Int J Environ Res Public Health. 2022 May 7;19(9):5690. doi: 10.3390/ijerph19095690 (PMC9100324; doi:10.3390/ijerph19095690)

Counteractive role of IL-33 and IL-37 in Osteoarthritis

Vikrant Rai[1], Matthew F. Dilisio[2], Farial Samadi[3], Devendra K. Agrawal[1*]

[1]Department of Translational Research, Western University of Health Sciences, Pomona, CA *91766*

[2] Department of Orthopedic Surgery, Creighton University School of Medicine, Omaha, NE 68178

[3]University of Nebraska at Omaha, Omaha, NE 68182

******Corresponding author***

Devendra K. Agrawal *Ph.D. (Biochem), Ph.D. (Med Sci), MBA, MS (ITM), FAAAAI, FAHA, FAPS, FIACS*

Senior Vice President for Research & Biotechnology

Professor, Department of Translational Research

Western University of Health Sciences

309 E. Second Street

Pomona, California 91766-1854, USA

Tel: Off: 909-469-7040; Fax: 909-469-5577

E-mail: DAgrawal@Westernu.edu

Running Title: Interleukin-33 and interleukin-37 in osteoarthritis

Supplementary Table S1: Primary antibodies used for immunofluorescence and flow cytometry. CD-cluster differentiation, CCR7-CC-chemokine receptor 7, GAPDH-glyceraldehyde 3-phosphate dehydrogenase, HMGB1- high mobility group box 1, IL interleukin, MMP- matrix metalloproteinases, NF-κB- nuclear factor-kappa beta, RAGE- receptor for advanced glycation end-products, TLRs-toll-like receptors, TNF α- tumor necrosis factor-alpha.

Target Protein	Antibody	Dilution
**For Immunofluorescence**		
IL-33	Rabbit anti-IL33 (sc-98659)	1:50
IL-37	Rabbit anti-IL37 (ab153889)	1:200
TLR-2	Goat anti-TLR2 (sc-8690)	1:50
TLR-4	Rabbit anti-TLR4 (sc-10741)	1:50
MyD88	Mouse anti-MyD88 (sc-136970)	1:50
IL-6	Goat anti-IL-6 (sc-1265)	1:50
TNFα	Goat anti-TNFα (sc-1348)	1:50
NF-κB	Rabbit anti-NF-κB (sc-372)	1:50
phospho NF-κB	Rabbit anti-phospho NF-κB (ab131109)	1:200
MMP-2	Mouse anti-MMP2 (sc-13595)	1:50
MMP-9	Goat anti-MMP9 (sc-6840)	1:50
Collagen II	Goat anti-collagen II (sc-389924)	1:50
Sox-9	Goat anti-sox-9 (sc-17340)	1:50
Chitinase-3 like protein 1	Rabbit anti-chitinase-3 (ab77528)	1:200
RAGE	Rabbit anti-RAGE (ab37647)	1:200

HMGB-1	Rabbit anti-HMGB1 (ab191583)	1:200
ST-2	Rabbit anti-ST2 (Millipore 06-1116)	1:100
CD14	Mouse anti-CD14 (ab182032)	1:200
CD86	Rabbit anti-CD86 (ab53004)	1:200
CD206	Rabbit anti-CD206 (ab64693)	1:200
CD163	Rabbit anti-CD163 (ab87099)	1:200
IL-10	Rat anti-IL-10 (JES3-19F1)	1:200
**For flow Cytometry**		
CD86	anti-mouse CD86 PE-Cyanin5 (isotype IgG2bκ PE-Cyanin5)	10µl/10^6
CCR7	anti-rat CCR7 APCeFluor780 (isotype IgG2aκ APCeFluor780)	10µl/10^6
CD206	anti-mouse CD206 Alexa Fluor 488 (isotype IgG1κ Alexa Fluor 488)	10µl/10^6
CD163	anti-mouse CD163 Per-CP-eFluor 710 (isotype IgG1κ Per-CP-eFluor 710)	10µl/10^6
IL-10	anti-mouse IL-10 PE-Cyanin 7 (isotype IgG1κ PE-Cyanin7)	10µl/10^6

Supplementary Table S2: Forward and reverse primer sequence of the gene of interest used for RT-PCR analysis. CD- cluster differentiation, GAPDH-glyceraldehyde 3-phosphate dehydrogenase, HMGB1- high mobility group box 1, IL interleukin, MMP- matrix metalloproteinases, NF-κB- nuclear factor-kappa beta, RAGE- receptor for advanced glycation end-products, TLRs-toll-like receptors, TNF α- tumor necrosis factor-alpha.

Gene of interest	Forward primer	Reverse primer
IL-33	5’- AAG CAC CCA GAT GCT TCA GT-3’	5’- TCC GCT TTT GCC ATA TCT TC -3’
IL-37	5’- CCT GCA GGG ACT CTT AGC TG -3’	5’- CTC CGA CTG CAG TGT GAA GA -3’
TLR-2	5’-CTG GAG AAA GCC TTG AAC TCT AT-3’	5’-GAC ACT CGG TCT CTA GCA ATT T-3’
TLR-4	5’-TCA AAG AGC TGG TGC GAA A -3’	5’-CAG CTG CTT GTC TGC ATT TG -3’
NF-κB	5’-GAC TAC GAC CTG AAT GCT GTG-3’	5’-GTC AAA GAT GGG ATG AGG AAG G-3’
IL-6	5’-ATA GGA CTG GAG ATG TCT GAG G-3’	5’-GCT TGT GGA GAA GGA GTT CAT AG-3’
TNF-α	5’-ACC CTC AAC CTC TTC TGG CTC AA-3’	5’-AAT CCC AGG TTT CGA AGT GGT GGT-3’
MMP-2	5’-TGATGGTGTCTGCTGGAAAG-3’	5’-CTACAGGACAGAGGGACTAGAG-3’
MMP-9	5’-ACAAGCTCTTCGGCTTCTG -3’	5’-GGTACAGGTCGAGTACTCCTTA-3’
HMGB-1	5’-AAG CAC CCA GAT GCT TCA GT-3’	5’- TCC GCT TTT GCC ATA TCT TC-3’
RAGE	5’- CCT GCA GGG ACT CTT AGC TG-3’	5’- CTC CGA CTG CAG TGT GAA GA-3’
CD14	5’-CTTGTGAGCTGGACGATGAA-3’	5’-TGCAGACACACACTGGAAG-3’
CD86	5’- AGG ACT CCC TCT AAG TGG AAT AG-3’	5’- GCC CAT AAG TGT GCT CTG AA-3’
CD206	5’-GGAGTGATGGTTCTCCTGTTTC-3’	5’- CCTTTCAGCTCACCACAGTATT-3’
GAPDH	5’- GGT GAA GGT CGG AGT CAA CGG ATT TGG TCG -3’	5’- GGA TCT CGC TCC TGG AAG ATG GTG ATG GG -3’

Supplementary Table S3: Flow-cytometry analysis of IL-33 and IL-37 treated macrophages (number (%) of positive cells). CD- cluster differentiation, IL- interleukin, SD- standard deviation. (N=3)

		CD86+ cells	CCR7+ cells	CD206+ cells	CD163+ cells
Control cells	Mean ± SD	1.77 ± 0.15	1.18 ± 0.025	0.82 ± 0.10	0.18 ± 0.07
IL-33 (10ng/ml)	Mean ± SD	85.1 ± 16.26	42.47 ± 6.44	0.72 ± 0.28	0.19 ± 0.02
	p value	<0.0001	<0.0001	0.999	0.999
IL-37 (10ng/ml)	Mean ± SD	2.36 ± 1.05	1.27 ± 0.20	**23.43** ± 5.41	**39.07** ± 9.81
	p value	0.9969	0.997	0.0003	0.0003
IL-37 (25ng/ml)	Mean ± SD			**33.8** ± 3.40	**64.9** ± 4.52
IL-37 (50ng/ml)	Mean ± SD			**31.3** ± 2.12	**60.8** ± 5.09

Supplementary Figure S1: Immunofluorescence for the characterization of chondrocytes in OA cartilage. Collagen II (Panel A), chitinase 3 like protein 1 (Panel D), Sox9 (Panel G), DAPI (Panels B, E, and H), and merged images (Panels C, F, and I). DAPI- 4, 6-diamidino-2-phenylindole. Panel J, K, and L- negative control for collagen II in cartilage. These are the representative images from all patients included in this study (N=3).

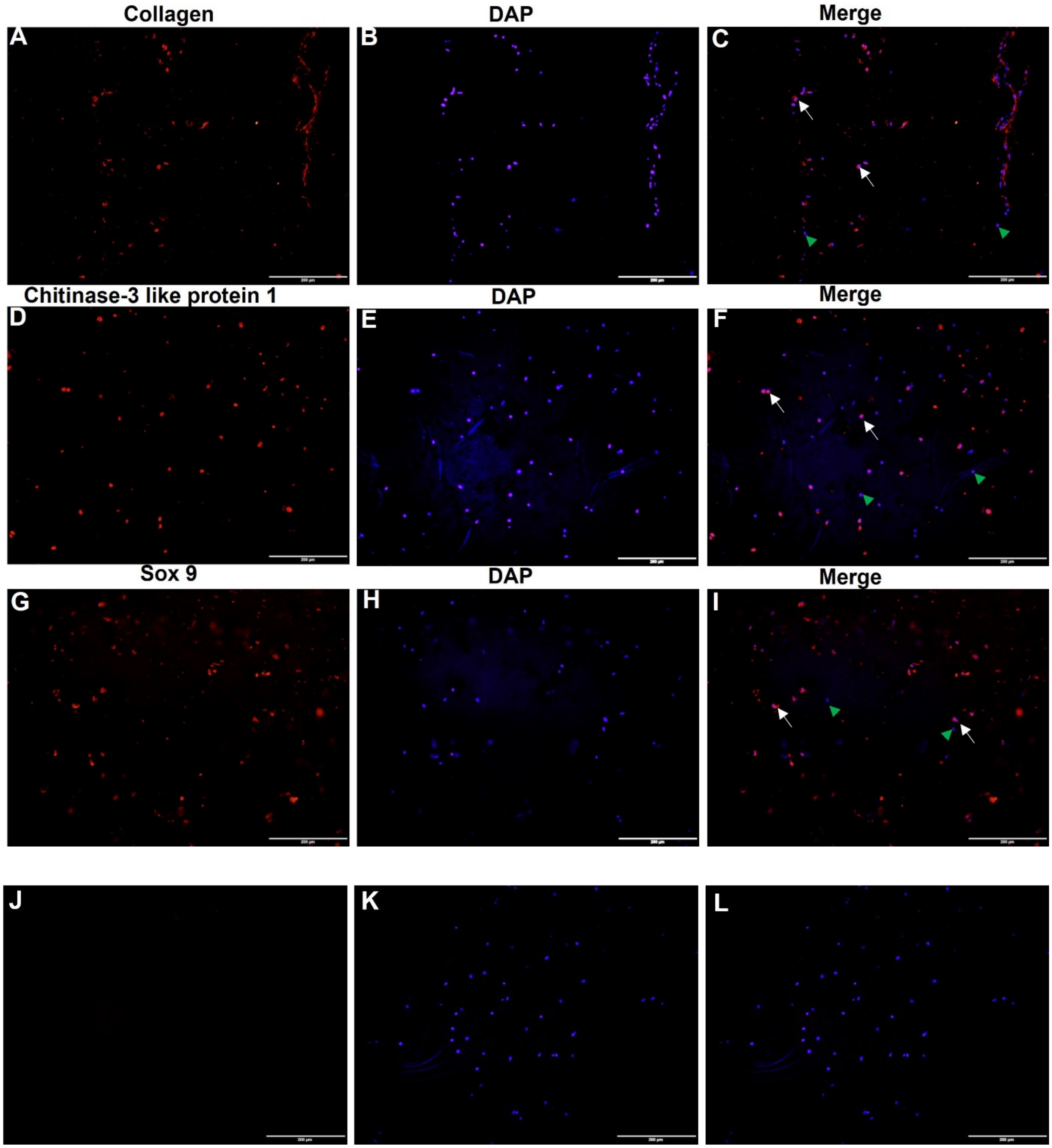

Figure S2: Immunofluorescence for the characterization of chondrocytes in NHAC and HCOA cells. Collagen II (Panels A and D), Sox9 (Panels G and J), chitinase 3 like protein 1 (Panels M and P), DAPI (Panels B, E, H, K, N, and Q), and merged images (Panels C, F, I, L, O, and R). DAPI- 4, 6-diamidino-2-phenylindole, HCOA- human chondrocytes osteoarthritis, NHAC- normal human articular chondrocytes. These are the representative images from three separate experiments (N=3). White arrows shows positive and green arrowheads shows negative immunostaining.

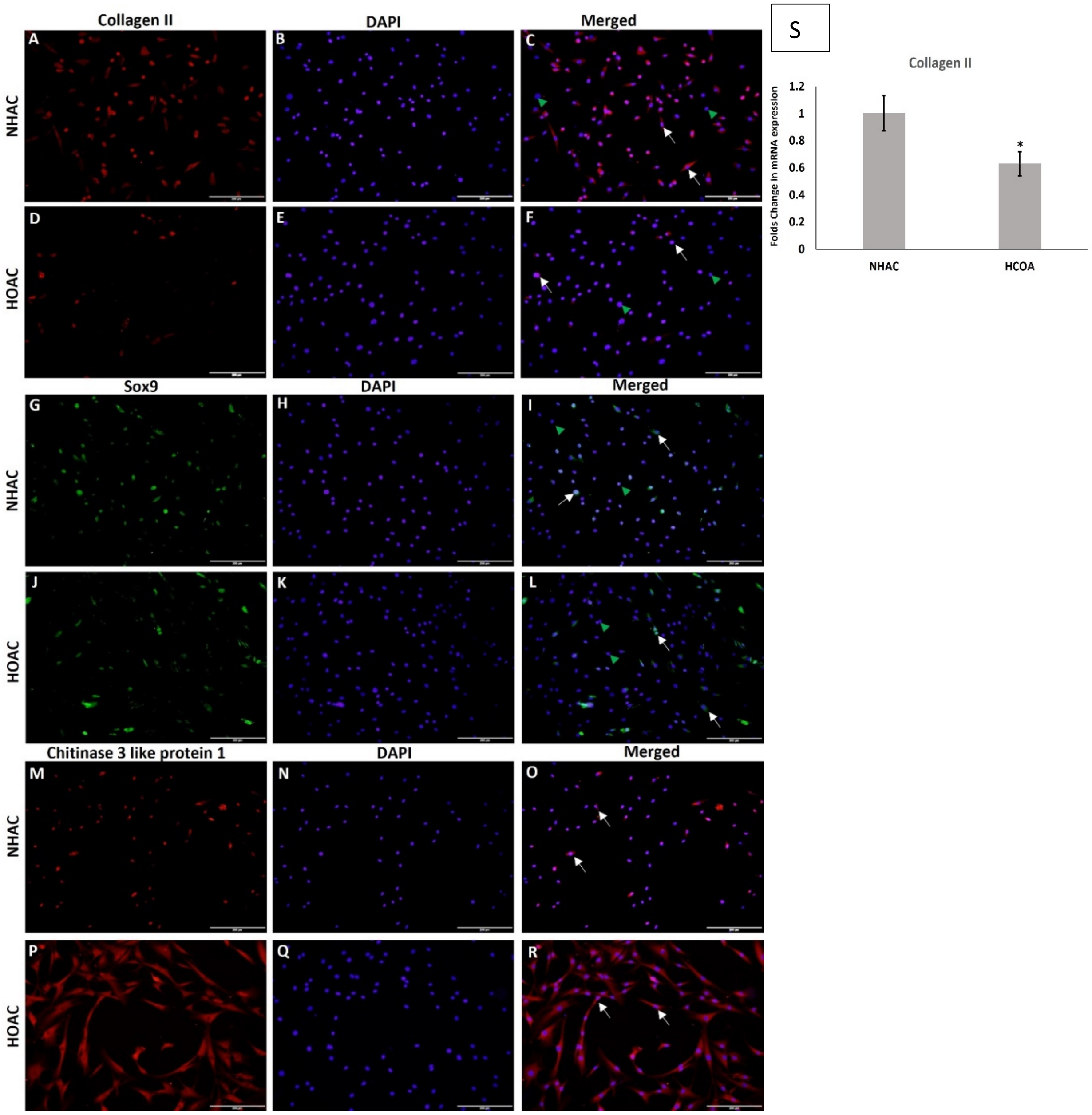

Supplementary Figure S3: Immunofluorescence for IL-6 and TNFα in OA knee and hip joint cartilage. IL-6 (Panels A and G), TNFα (Panels D and J), DAPI (Panels B, E, H, and K), and merged images (Panels C, F, I, and L). DAPI- 4, 6-diamidino-2-phenylindole, OA- osteoarthritis, IL6-interleukin 6, TNFα-tumor necrosis factor-alpha. These are the representative images from all patients included in this study

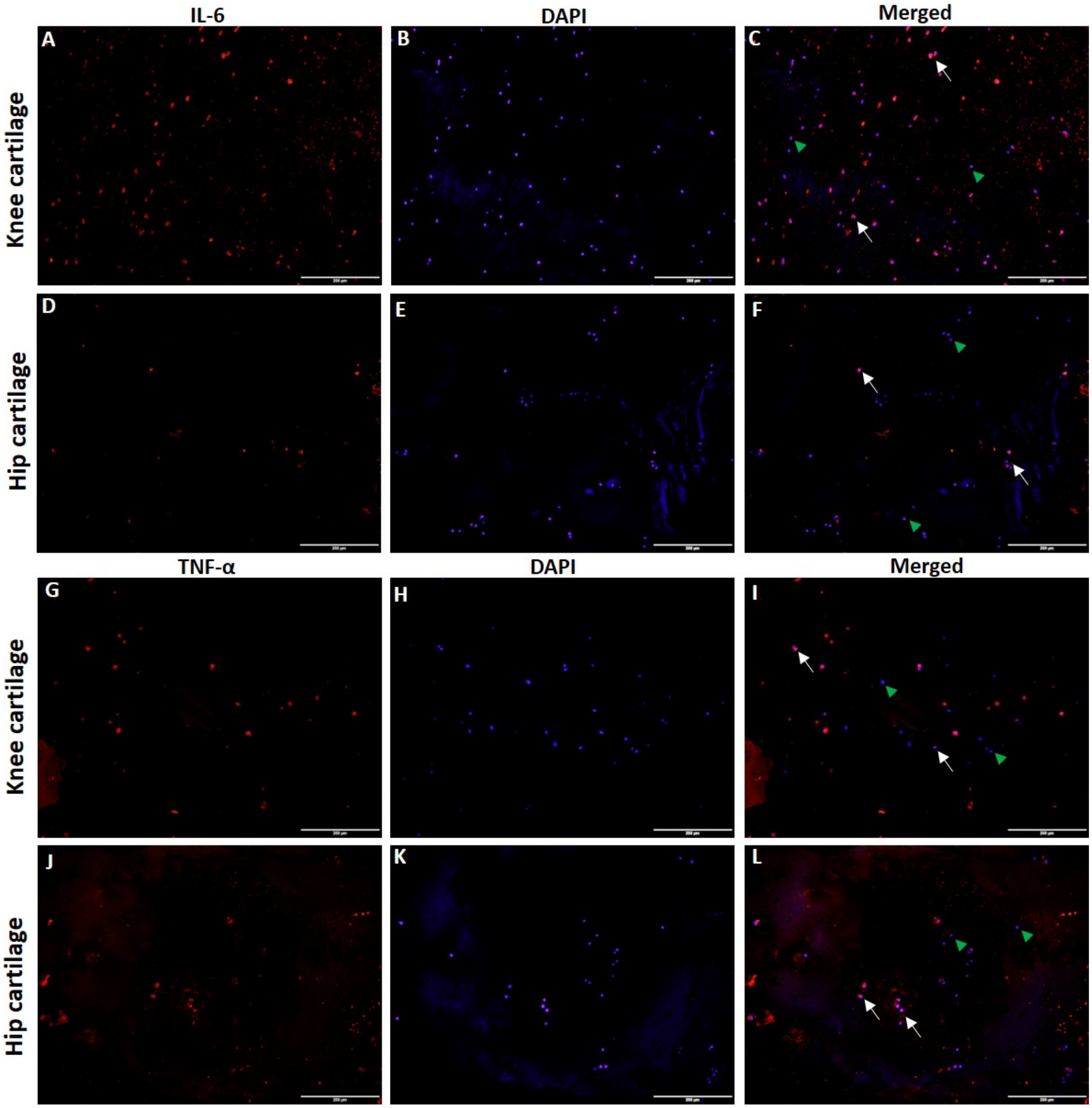

Supplementary Figure S4: Immunofluorescence for MMP2 and MMP9 in OA cartilage and fat tissue: MMP2 (Panels A, G, and M), MMP9 (Panels D, J, and P), DAPI (panels B, E, H, K, N, and Q), merged images (Panels C, F, I, L, O, and R). DAPI- 4, 6-diamidino-2-phenylindole, MMP-matrix metalloproteinases. These are the representative images from all patients included in this study (N=3).

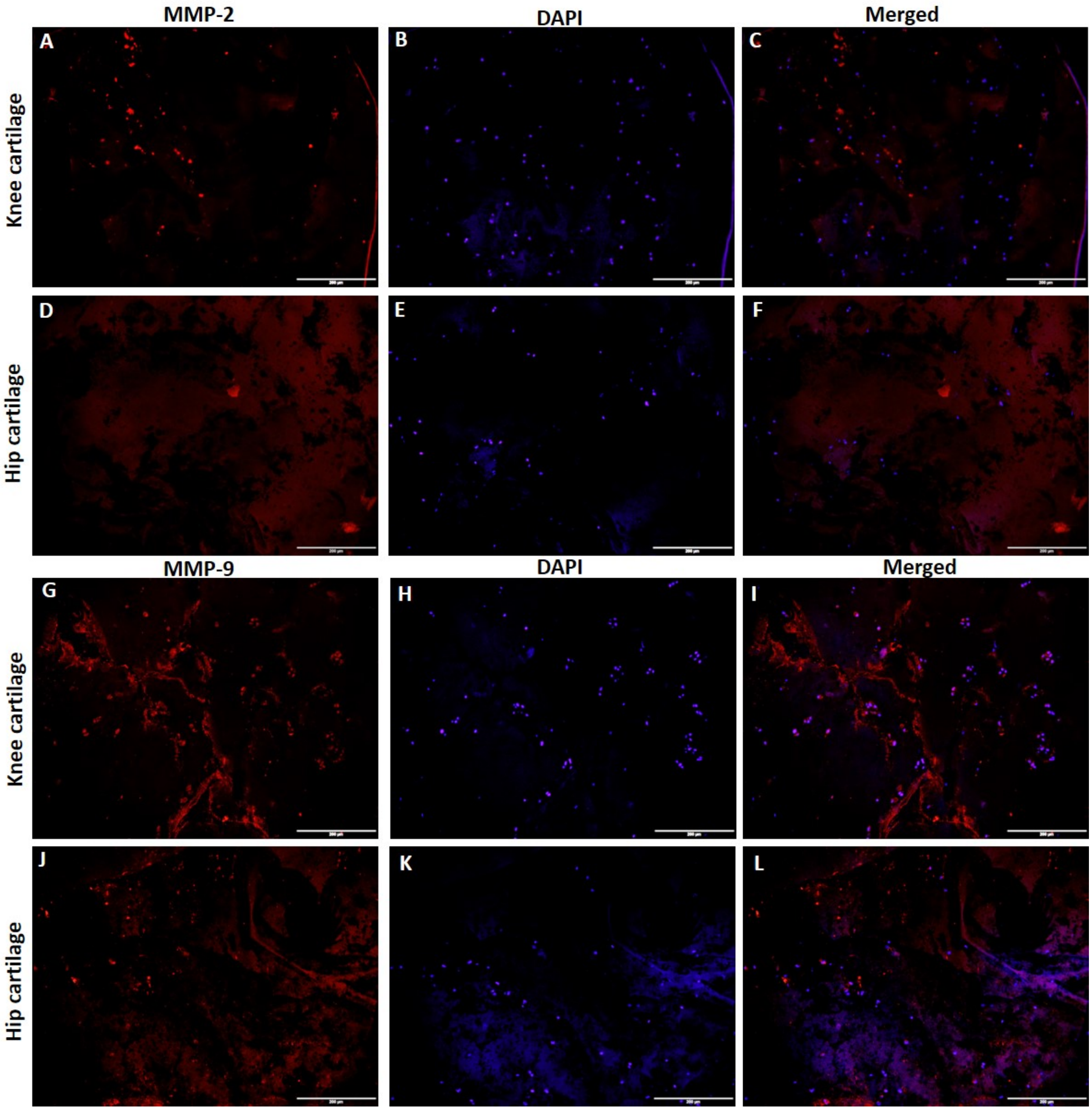

Supplementary Figure S5: Immunofluorescence for NF-κB in OA cartilage. P^{50} NF-κB (Panel A), p^{65} NF-κB (Panel D), DAPI (Panels B and E), and merged images (Panels C and F). DAPI- 4, 6-diamidino-2-phenylindole, NF-κB- nuclear factor kappa beta, OA-osteoarthritis. These are the representative images from all patients included in this study.

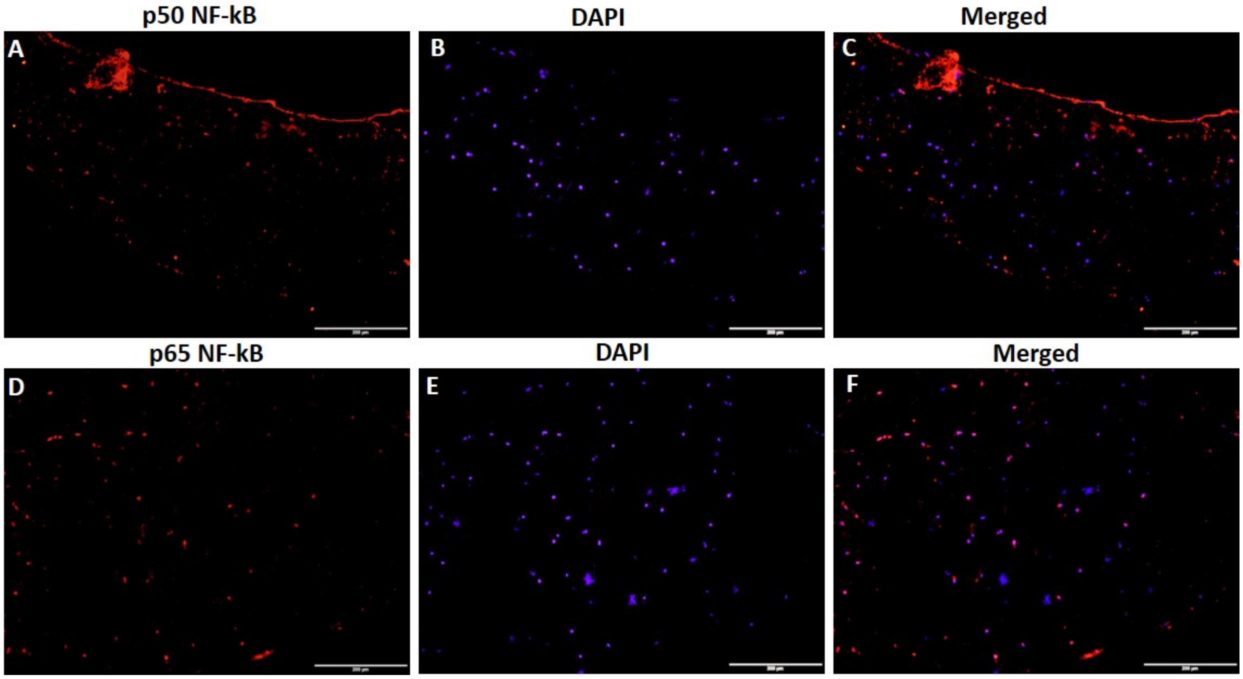

Supplementary Figure S6: Immunofluorescence for IL-33 and IL-37 in NHAC and HCOA cells. IL-33 (Panels A and E), IL-37 (Panels I and L), ST2 (Panels B and F), DAPI (Panels C, G, J, and M), merged image (Panels D, H, K, and N); DAPI-4, 6-diamidino-2-phenylindole, HCOA- human osteoarthritic chondrocytes, IL-interleukin, NHAC- normal human articular chondrocytes. These are the representative images from three separate experiments.

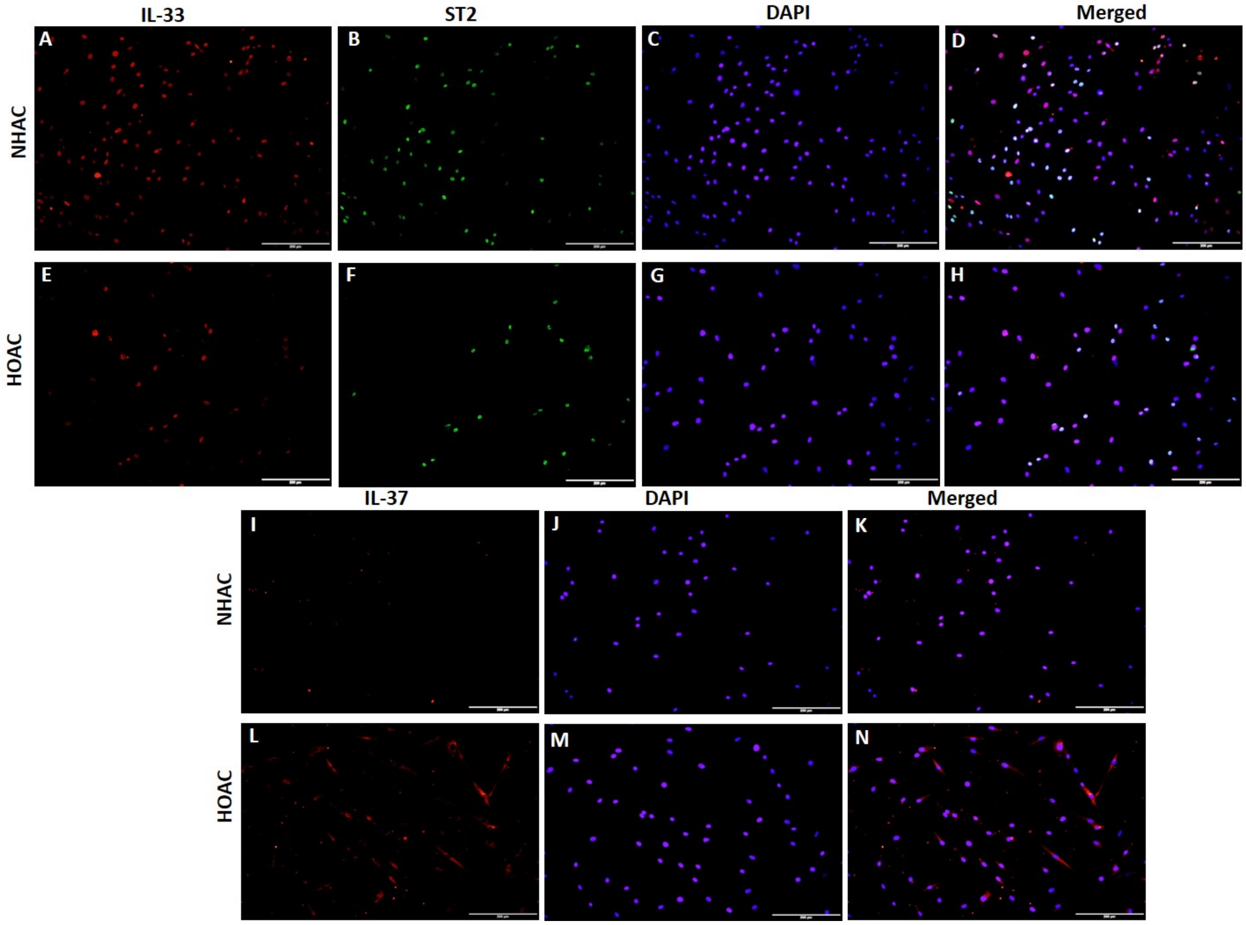

Supplementary Figure S7: Immunofluorescence for TLR2/MyD88 and TLR4/MyD88 in NHAC and HCOA cells. TLR2 (Panels A and E), TLR4 (Panels I and M), MyD88 (B, F, J, and N), DAPI (Panels C, G, K, and O), merged image (Panels H, H, L, and P); DAPI- 4, 6-diamidino-2-phenylindole, HCOA- human osteoarthritic chondrocytes, MFI-mean fluorescence intensity, MyD88- myeloid differentiation primary response 88, NHAC- normal human articular chondrocytes, TLR- toll-like receptor. These are the representative images from three separate experiments.

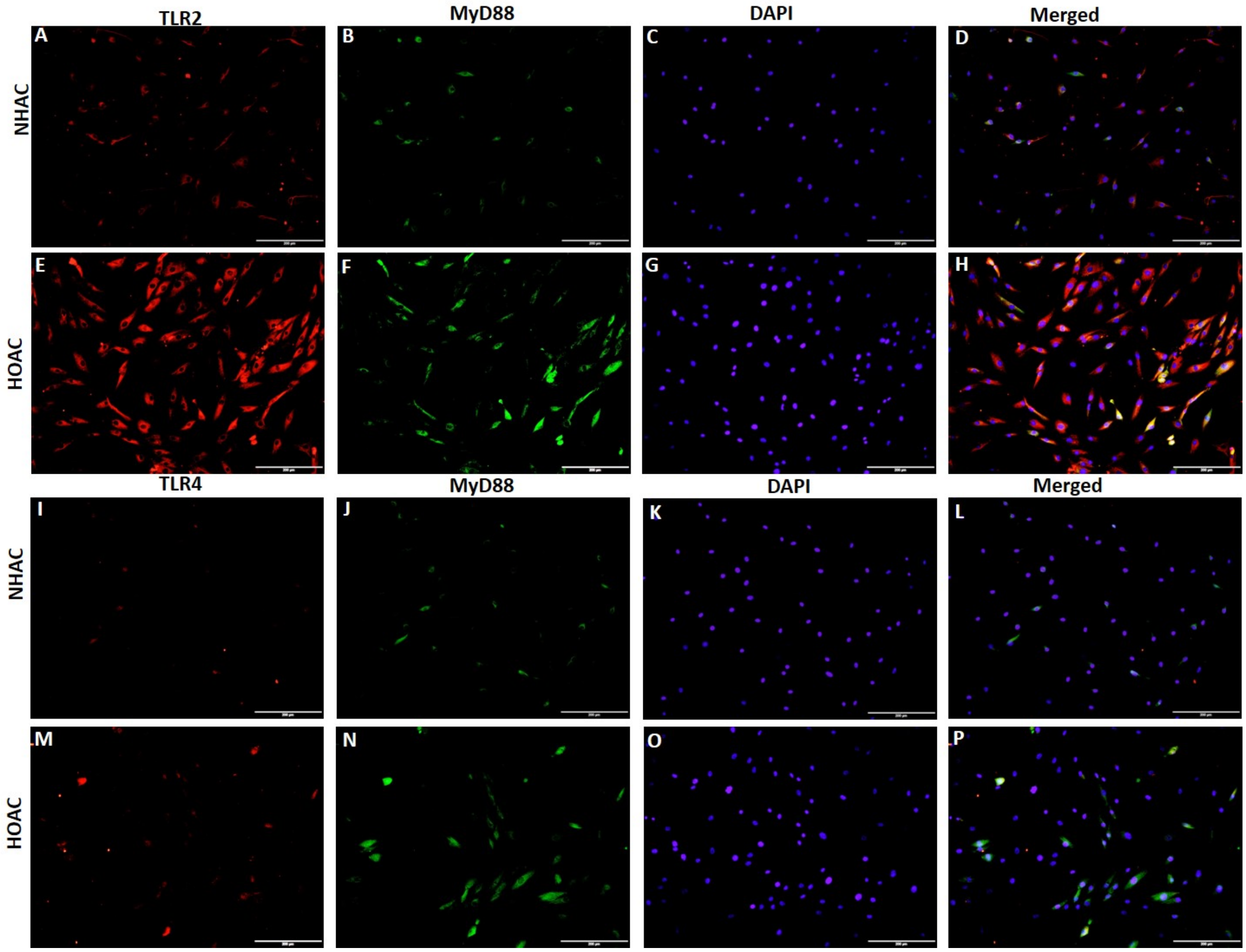

Supplementary Figure S8: Immunofluorescence for IL-6 and TNFα in NHAC and HCOA cells. IL-6 (panels A and D), TNFα (panels G and J), , DAPI (Panels B, E, H, and K), and merged images (Panels C, F, I, and L). DAPI- 4, 6-diamidino-2-phenylindole, HCOA- human chondrocytes osteoarthritic, IL6-interleukin 6, NHAC- normal human articular chondrocytes, TNFα- tumor necrosis factor-alpha. These are the representative images from three separate experiments.

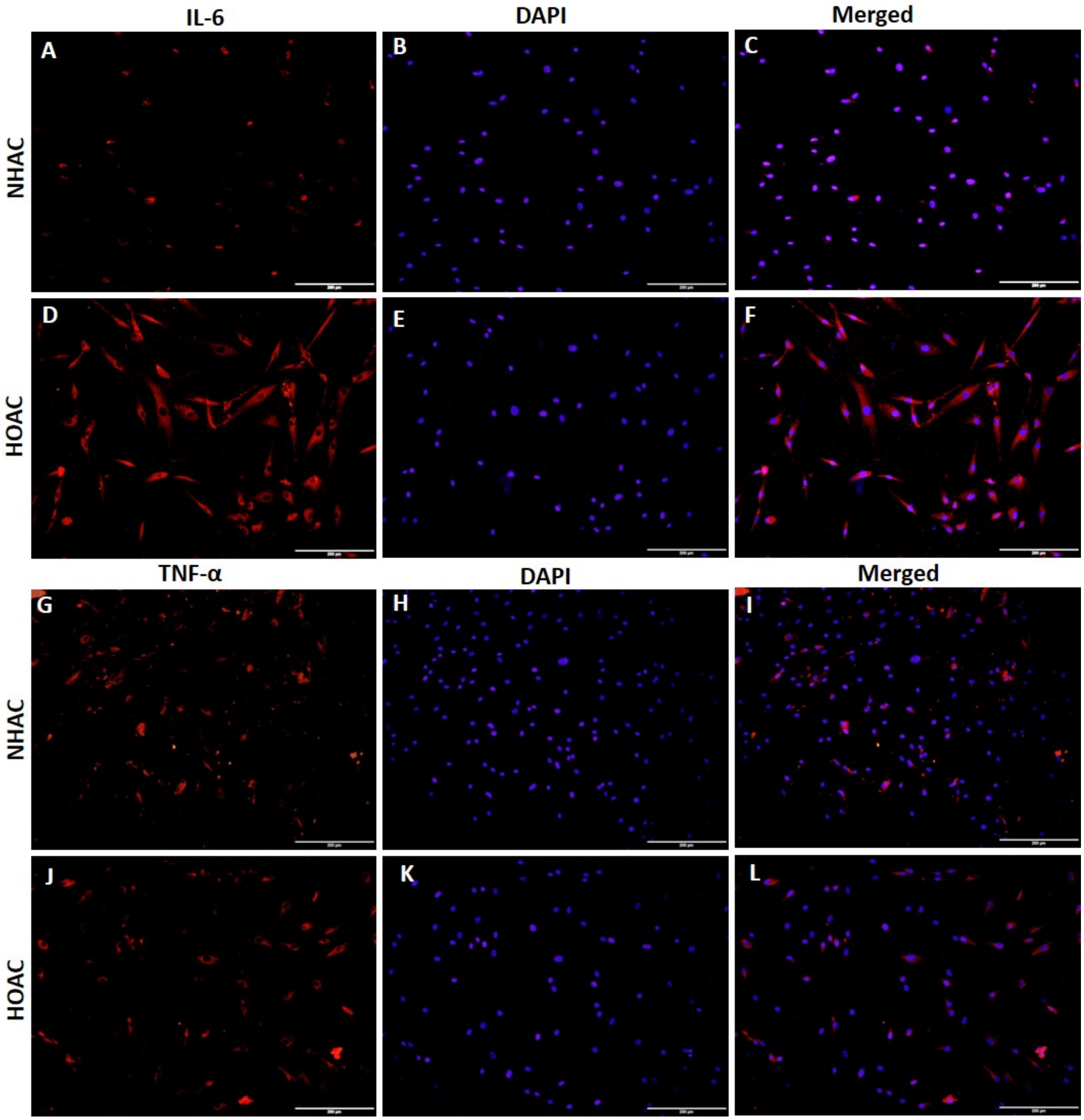

Supplementary Figure S9: Immunofluorescence for MMP2 and MMP9 in NHAC and HCOA cells. MMP2 (Panels A and D), MMP9 (Panels G and J), DAPI (Panels B, E, H, and K), and merged images (Panels C, F, I, and L). DAPI- 4, 6-diamidino-2-phenylindole, HCOA- human chondrocytes osteoarthritic, MMP- matrix metalloproteinases, NHAC- normal human articular chondrocytes. These are the representative images from three separate experiments (N=3).

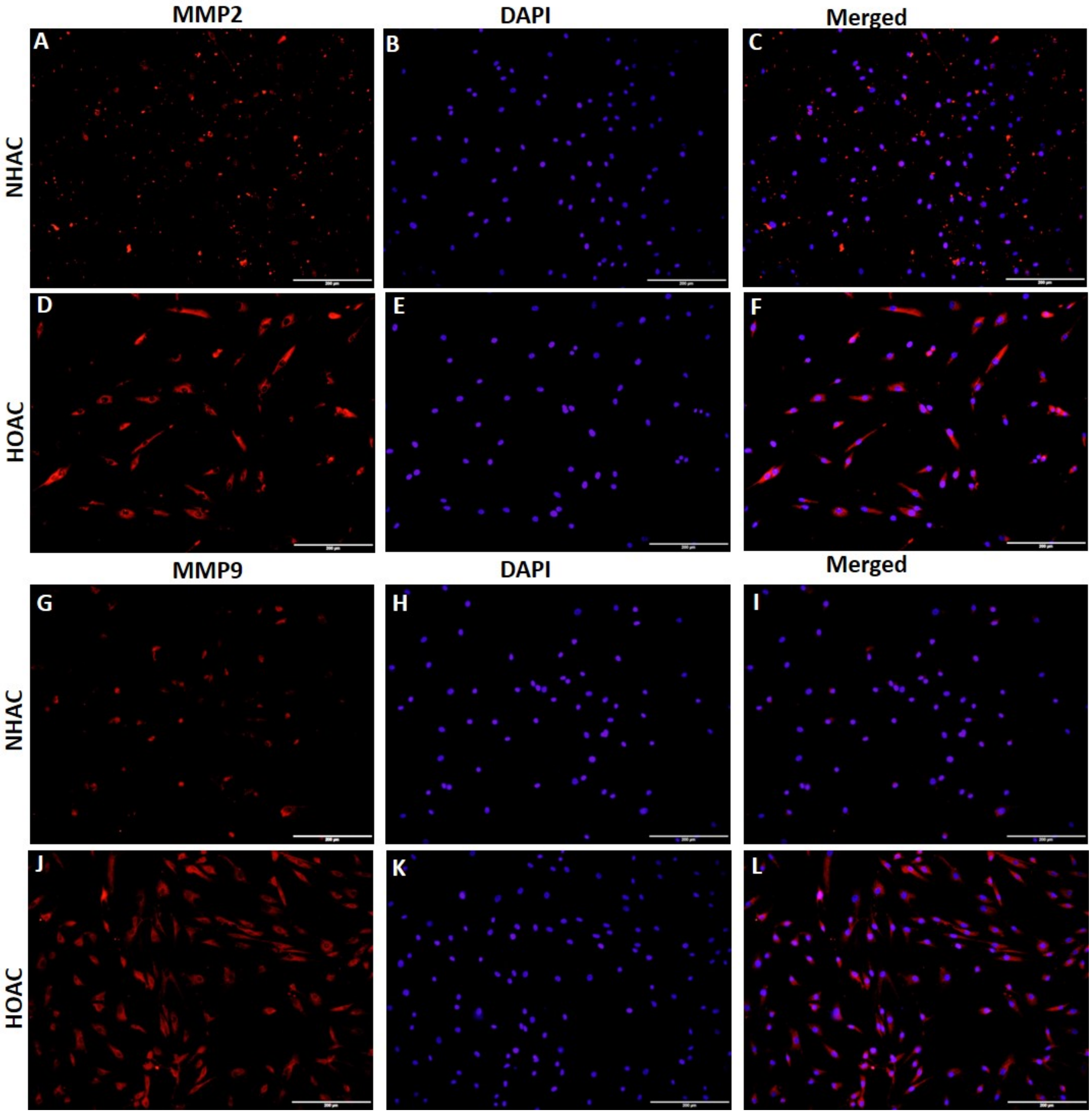

Supplementary Figure S10: Immunofluorescence for NF-κB NHAC and HCOA cells. NF-κB (Panels A, D, G, and J), DAPI (Panels B, E, H, and K), and merged images (Panels C, F, I, and L). DAPI- 4, 6-diamidino-2-phenylindole, HCOA- human chondrocytes osteoarthritic, NF-κB- nuclear factor kappa beta, NHAC-normal human articular chondrocytes. These are the representative images from three separate experiments (N=3).

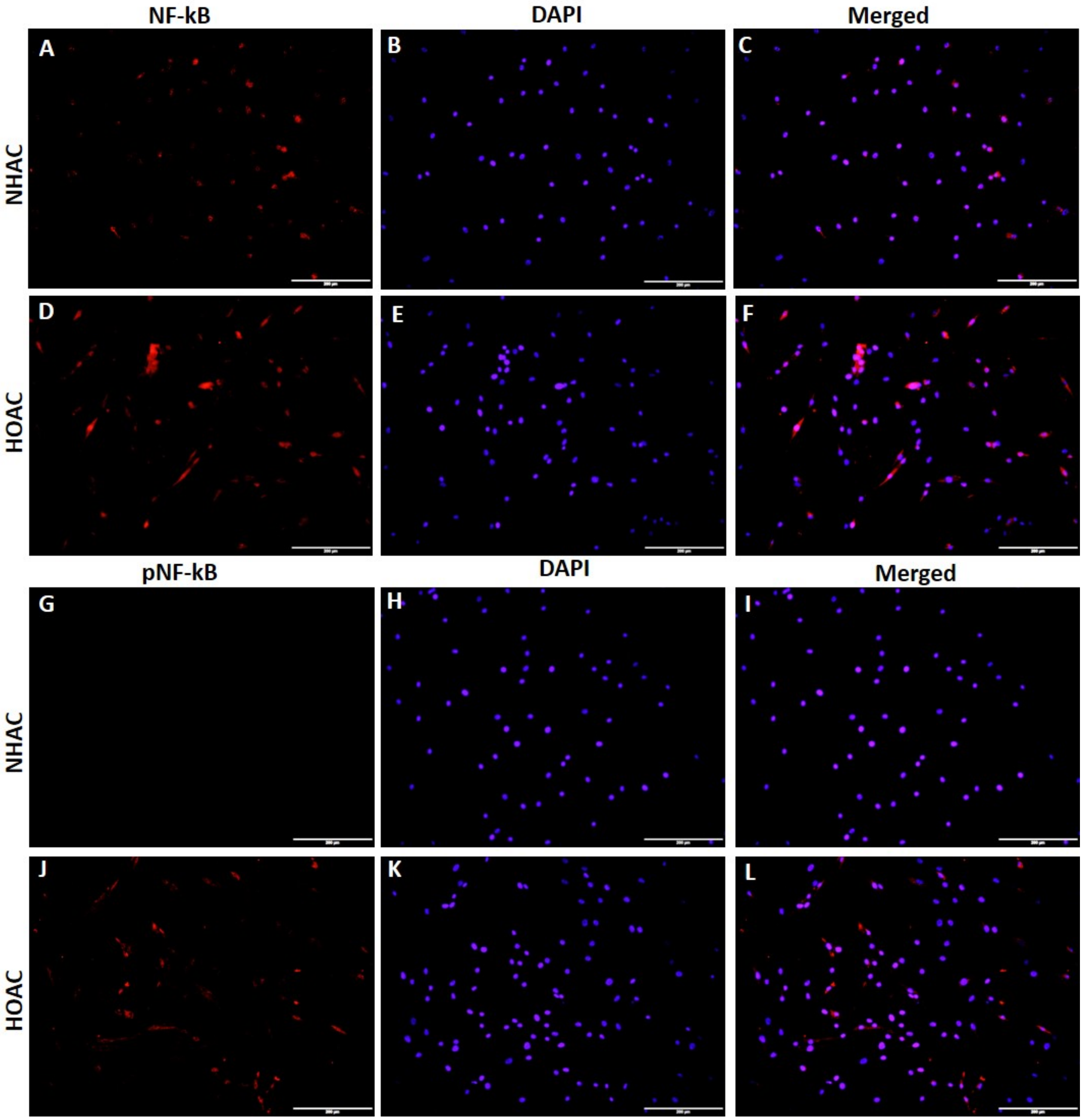

Supplementary Figure S11: Immunofluorescence of macrophage in OA cartilage: Dual fluorescence staining of the tissue from the osteoarthritic joint for CD14 (panels A, E, I, and M) with CD163 (panels B and F), and IL-10 (panels J and N) showed minimal to no immunopositivity.

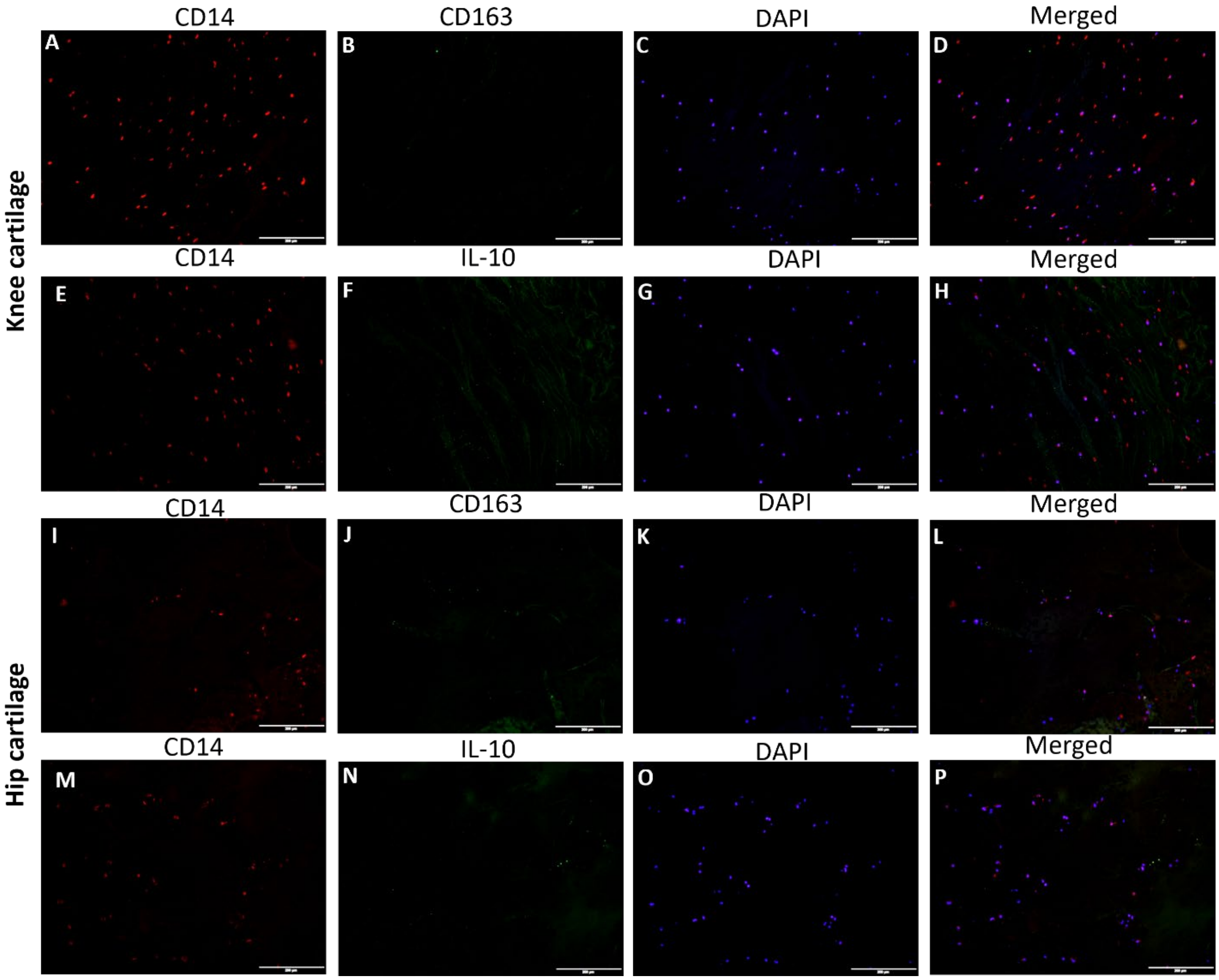

Supplementary Figure S12: Immunofluorescence staining for the co-localization of TLR-2, TLR-4, and RAGE with HMGB-1: Dual immunofluorescence studies for TLR-2, TLR-4, and RAGE with HMGB-1 showed the co-localization of HMGB-1 with TLR-2, TLR-4, and RAGE. Co-localization of HMGB-1 with TLRs and RAGE suggests that HMGB-1can stimulate both on these cells and RAGE is a ligand for HMGB-1

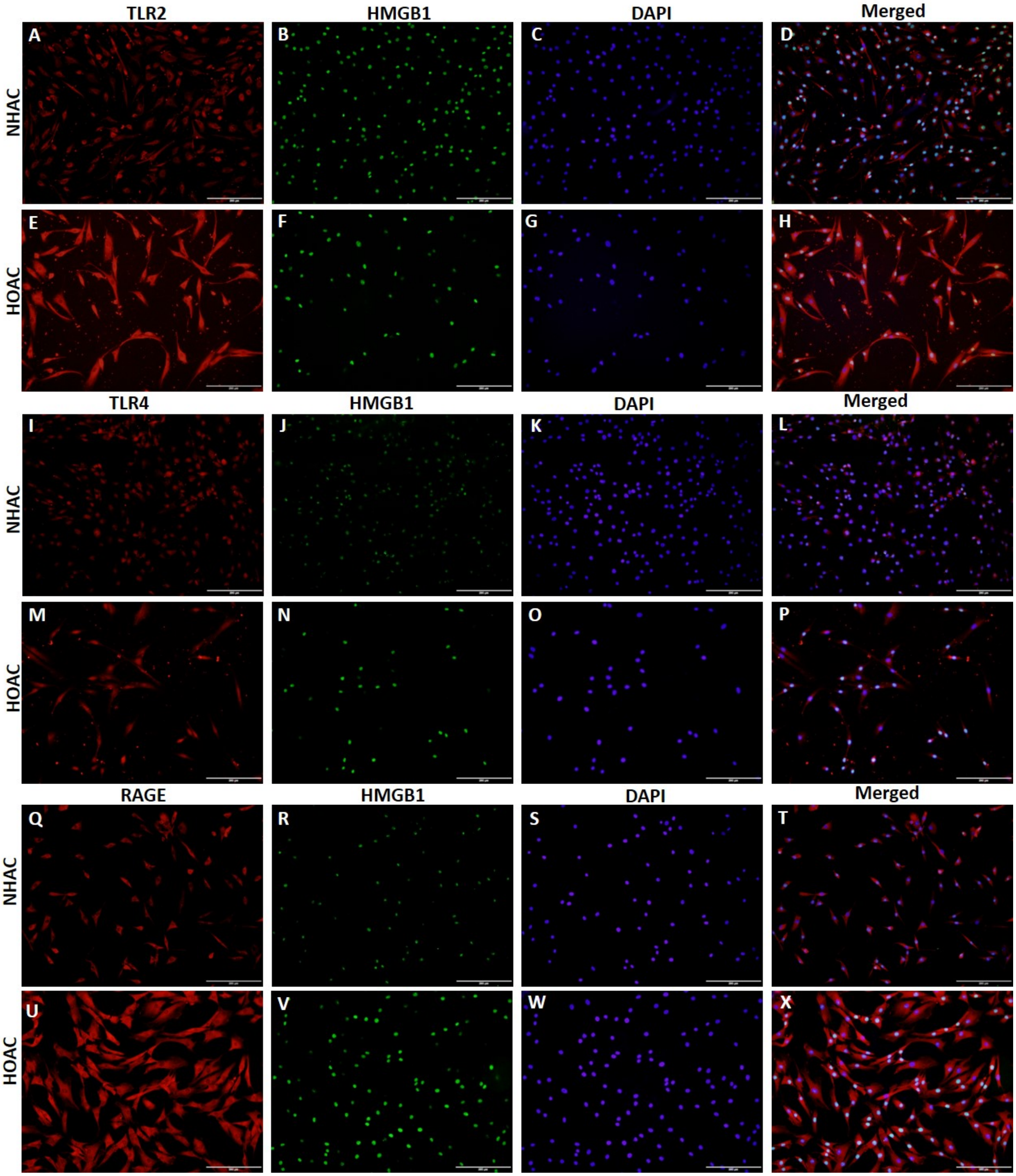

Supplementary Figure S13: Immunofluorescence of CCR2, CCL3, CCL5, and CCR7 in osteoarthritic human cartilage: Immunofluorescence of osteoarthritic human knee cartilage revealed immunopositivity for CCR2, CCL3, CCL5, and CCR7. The immunopositivity for these suggests the presence of chemokine receptors and ligands specific for macrophage and monocytes in the osteoarthritic human cartilage.

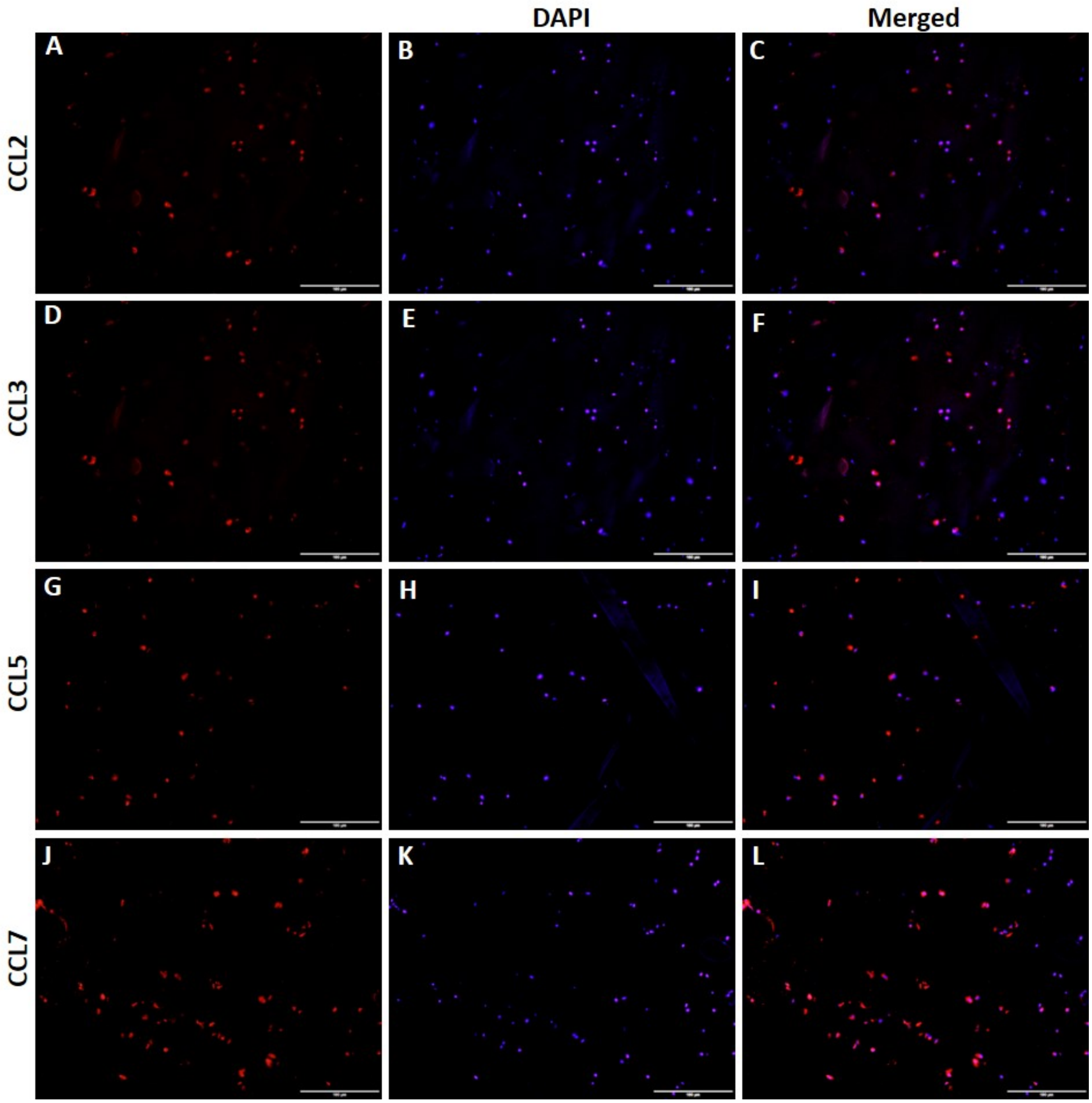

Supplementary Figure S14: Immunopositivity for vasculoendothelial growth factor (VEGF) and CD31 in human osteoarthritic cartilage: Immunofluorescence of osteoarthritic human cartilage revealed immunopositivity for VEGF and CD31. Immunofluorescence also revealed immunopositivity for VEGF in degenerating swine cartilage. The immunopositivity for CD31 suggests the presence of endothelial cells and the immunopositivity for VEGF suggests the presence of growth factor essential for vasculogenesis.

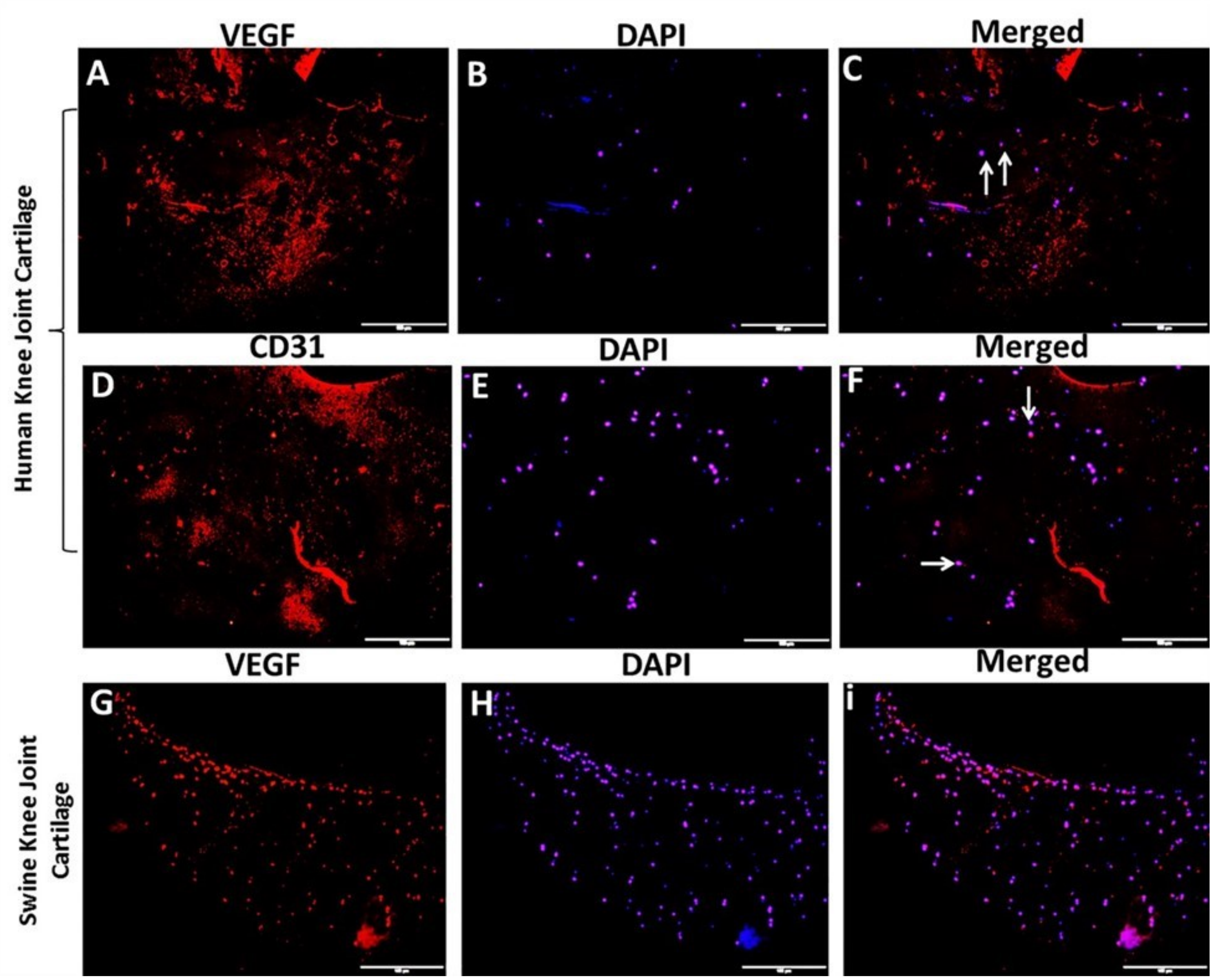

Supplementary Figure S15: Immunofluorescence of macrophage in OA joint fat: Dual fluorescence staining of the fat tissue from the osteoarthritic joint for CD14 (panels A, E, I, and M) with CD86 (panels B and F), and CD206 (panels J and N) showed immunopositivity for CD14+CD86+cells (M1 macrophages), CD14+CD206+ cells (M2a macrophages); DAPI panels C, G, K, and O) and merged images (panels D, H, L and P); mean fluorescence intensity of CD14+CD86+ and CD14+CD206+cells (panel Q) and macrophage density (panel R) in the knee and hip joint. The data has been represented as average ± SD. A $p<0.05$ was considered significant. ***$p<0.001$ and ****$p<0.0001$.

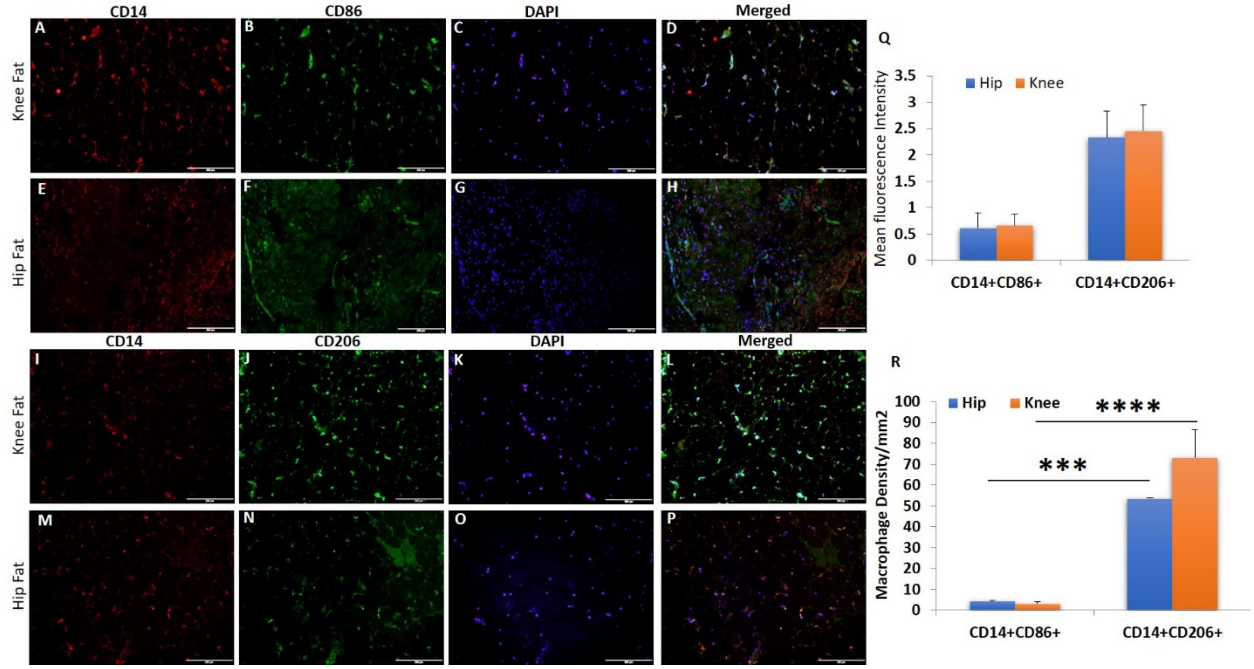

Supplement: Supplementary file 1 [file ijerph-19-05690-s001.zip › ijerph-1672361-supplementary.pdf]
